# MRGPRX2-Mediated Degranulation of Human Skin Mast Cells Requires the Operation of G_αi_, G_αq_, Ca++ Channels, ERK1/2 and PI3K—Interconnection between Early and Late Signaling

**DOI:** 10.3390/cells11060953

**Published:** 2022-03-10

**Authors:** Zhao Wang, Kristin Franke, Gürkan Bal, Zhuoran Li, Torsten Zuberbier, Magda Babina

**Affiliations:** 1 Institute for Allergology, Charité—Universitätsmedizin Berlin, Corporate Member of Freie Universität Berlin, Humboldt-Universität zu Berlin, and Berlin Institute of Health, 10117 Berlin, Germany; wang.zhao@xjtu.edu.cn (Z.W.); kristin.franke@charite.de (K.F.); guerkan.bal@charite.de (G.B.); zhuoran.li@charite.de (Z.L.); torsten.zuberbier@charite.de (T.Z.); 2 Department of Dermatology, The Second Affiliated Hospital, Northwest Hospital, Xi’an Jiaotong University, Xi’an 710004, China; 3 Fraunhofer Institute for Translational Medicine and Pharmacology ITMP, Allergology and Immunology, 12203 Berlin, Germany

**Keywords:** mast cells, MRGPRX2, FcεRI, degranulation, G proteins, signal transduction, MAP kinases, ERK1/2, PI3K/AKT, skin

## Abstract

The recent discovery of MRGPRX2 explains mast cell (MC)-dependent symptoms independently of FcεRI-activation. Because of its novelty, signaling cascades triggered by MRGPRX2 are rudimentarily understood, especially in cutaneous MCs, by which MRGPRX2 is chiefly expressed. Here, MCs purified from human skin were used following preculture or ex vivo and stimulated by FcεRI-aggregation or MRGPRX2 agonists (compound 48/80, Substance P) in the presence/absence of inhibitors. Degranulation was assessed by β-hexosaminidase or histamine release. Phosphorylation events were studied by immunoblotting. As a G protein-coupled receptor, MRGPRX2 signals by activating G proteins; however, their nature has remained controversial. In skin MCs, G_αi_ and G_αq_ were required for degranulation, but G_αi_ was clearly more relevant. Ca++ channels were likewise crucial. Downstream, PI3K was essential for granule discharge initiated by MRGPRX2 or FcεRI. ERK1/2 and JNK were additional participants, especially in the allergic route. Addressing possible points of intersection between early and later events, pERK1/2 and pAKT were found to depend on G_αi_, further highlighting its significance. G_αq_ and Ca++ channels made some contributions to the phosphorylation of ERK. Ca++ differentially affected PI3K activation in FcεRI- vis-à-vis MRGPRX2-signaling, as channel inhibition increased pAKT only when triggered via FcεRI. Collectively, our study significantly extends our understanding of the molecular framework behind granule secretion from skin MCs.

## 1. Introduction

With the discovery of Mas-related G protein-coupled receptor member X2 (MRGPRX2) as a key receptor of IgE-independent mast cell (MC) degranulation, two similarly effective routes of degranulation are now established in skin MCs [1,2]. The presumed clinical significance of MRGPRX2 stems from the plethora of ligands, both endogenous and exogenous, that reportedly activate the receptor [1,3,4,5,6,7,8,9].

Since MRGPRX2 was discovered fairly recently, signaling cascades elicited by its ligands are still ill-defined. As a G protein-coupled receptor (GPCR), MRGPRX2 initiates signaling by activation of G proteins. Which G protein subsets are involved is not completely clear, since pertussis toxin (PTX), a G_αi_ inhibitor [10,11]), and YM-254890, that is G_αq_ specific [12], have been variably shown to interfere with MRGPRX2 signaling [13,14,15]; it is assumed that the cell type expressing MRGPRX2 plays a decisive role, but no data are currently available for skin MCs. However, cutaneous MCs are not only the most abundant MC population in the body [16], but they also express the highest levels of MRGPRX2 and respond most vigorously to its ligands [17,18,19,20]. In addition, skin MCs critically contribute to acute hypersensitivity reactions and anaphylaxis [21,22,23,24].

We recently reported that, in addition to degranulation, MRGPRX2 can elicit cytokine responses in skin MCs, yet less efficiently than the canonical high-affinity IgE receptor (FcεRI)-dependent route. Interestingly, we also found that the two systems stimulate the activity of kinases with inverted kinetics (MRGPRX2 quickly, FcεRI slowly), while a dominant ERK1/2 module was revealed for both receptors. ERK1/2 was also functionally the most significant cascade to drive cytokine production.

Notwithstanding, cytokines are rather implicated in chronic conditions, while it is degranulation that will drive acute reactions like MC-dependent hypersensitivity, urticaria and anaphylaxis. In MCs, the signaling processes leading to cytokine generation typically differ from those facilitating granule exocytosis, and especially ERK activity, which is crucial in the former but assumed not to be in the latter [25,26,27,28].

Herein, we, therefore, set out to identify the signaling prerequisites underlying skin MC degranulation by the novel route in comparison with the canonical IgE-dependent pathway. Since it was unresolved how early (G protein activation, Ca++ channels) and later signaling components (kinase activity) are interconnected, we addressed possible points of intersection. Unexpectedly, we identify ERK1/2 as a crucial element of the degranulation machinery of skin MCs not only for the MRGPRX2-triggered route but also for the FcεRI-dependent pathway. Focusing on the interdependence of early and late signaling events following MRGPRX2 ligation, we identify interesting interconnections, and find differences with respect to the FcεRI-driven pathway. In essence, this study makes important contributions regarding the molecular underpinnings driving the degranulation of skin MCs.

## 2. Materials and Methods

### 2.1. Skin MC Purification and Culture

Foreskins were obtained from circumcisions with the written informed consent of the patients or their legal guardians, as described in previous studies [29,30,31]. The study was approved by the Ethics Committee of the Charité Universitätsmedizin Berlin and experiments were conducted according to the Declaration of Helsinki Principles.

MCs were isolated from skin samples as described by a routine procedure [32,33]. Briefly, foreskin samples (normally pooled from 2–10 donors) were cut into strips and treated with dispase (BD Biosciences, Heidelberg, Germany) 4 °C overnight at 3.5 U/mL. The epidermis was removed; the dermis was then chopped to homogeneity and digested with an enzyme cocktail containing 1.5 mg/mL of collagenase type 1 (Worthington, Lakewood, NJ, USA), 0.75 mg/mL hyaluronidase type 1-S (Sigma, Steinheim, Germany), and DNase I at 10 μg/mL (Roche, Basel, Switzerland) at 37 °C in a shaking water bath for 75 min. The cells were separated from the remaining tissue by filtration and incubated with anti-human c-Kit microbeads for positive selection by the Auto-MACS (both from Miltenyi Biotec, Bergisch Gladbach, Germany). MC purity (>98%) was assessed by toluidine blue staining and viability (>99%) by trypan blue. Purified skin MCs were either used ex vivo (within 18 h) or cultured in Basal Iscove’s medium with 10% FCS (Biochrom, Berlin, Germany) at the concentration of 5 × 10^5^/mL, with SCF (at 100 ng/mL) (Peprotech, Rocky Hill, NJ, USA) and IL-4 (at 10 ng/mL) (Peprotech) was provided twice a week. The culture time was 2–4 weeks.

### 2.2. MC Treatment

For G proteins and Ca++ channel inhibition, cells were pretreated for 16 h with PTX at 200 ng/mL (G_αi_ inhibitor, List Biological Labs, Campbell, CA, USA), or with YM-254890 at 10 µM (G_αq_ inhibitor, Fujifilm, Osaka, Japan), 2-Aminoethyl diphenylborinate at 100 µM (2-APB, a dual inhibitor of inositol 1,4,5-trisphosphate receptor, IP3R, and Orai-1/Orai-2, Santa Cruz Biotechnology, Dallas, TX, US), or Lanthanum (III) chloride heptahydrate at 1 µM (La3+, a Ca++ release-activated channel blocker, Sigma-Aldrich, Taufkirchen, Germany). Pre-treatment with the latter inhibitors was for 5 min. The inhibitors, their primary targets and relevant literature are specified in Supplementary Table S1.

For kinase inhibition, cells were pre-incubated with SP600125 (JNK inhibitor, 5 µM, ApexBio, Houston, TX, USA), pictilisib (PI3K inhibitor, 5 µM, Selleckchem, München, Germany), SCH772984 (ERK1/2 inhibitor, 10 µM, BioVision, Milpitas, CA, USA) or Vx-11e (ERK2 inhibitor, 2 µM, BioVision, Milpitas, CA, USA) 15 min prior to downstream analyses.

Toxicity of the inhibitors was assessed by viability and apoptosis assays using the Zombie Aqua (ZA) Viability kit (BioLegend, San Diego, CA, USA) and the Vybrant™ apoptosis assay kit #4 (Molecular Probes, Eugene, OR, USA). The toxicity of the compounds was measured on cultured and freshly isolated (ex vivo) mast cells. Briefly, mast cells were incubated for one hour with the inhibitor or with the inhibitor solvent alone. Cells were then washed twice with PBS and stained separately with the apoptosis kit and viability kit according to the manufacturer’s instructions. Since PTX requires longer preincubation times, an additional staining was performed for PTX-incubated cells after 24 h. After staining, cells were immediately analyzed using the MACSQuant^®^ Analyzer 10 flow cytometer (Miltenyi Biotec Inc., Auburn, CA, USA). The inhibitors showed comparable viability and percentage of apoptotic cells compared with the corresponding controls (Supplementary Figures S1 and S2).

### 2.3. Histamine Release Assay (HRA)

The HRA was performed according to a method routinely employed in our laboratory [34,35]. In brief, MCs pretreated with or without inhibitors were stimulated by FcεRI-aggregation. The anti-AER-37 antibody (eBioscience, San Diego, CA, USA) was used for cultured MCs at 0.1 µg/mL, and the anti-FcεRIα-Ab 29C6 (kind gift from Dr. Hakimi, Hoffmann La Roche, Nutley, NJ, USA) at 0.5 µg/mL served for the stimulation of ex vivo MCs. MRGPRX2 stimulation was achieved by compound 48/80 (c48/80, Sigma, at 10 µg/mL), or substance p (SP, Bachem, Budendorf, Switzerland at 30 µM). Spontaneous release was determined in the absence of any stimulus. Assays were performed in PAG-CM buffer (Piperazine-N,N-bis [2-ethanesulfonic acid]-Albumin-Glucose buffer containing 3 mM CaCl_2_ and 1.5 mM MgCl_2_, pH 7.4) for 30 min at 37 °C. Histamine in the supernatants was measured by an automated fluorescence method (Alliance Instruments, Salzburg, Austria). Total cellular histamine content was measured analogously. All determinations were performed in triplicate. Net histamine release (%) was calculated as [(stimulated release–spontaneous release)/complete histamine in the MC preparation] × 100.

### 2.4. β-Hexosaminidase Release Assay

Detection of β-hexosaminidase was performed as described [30,33]. Briefly, cells were pretreated with inhibitors, then challenged by the stimuli for 60 min in PAG-CM buffer as described under histamine release. Supernatants (SNs) were collected, and the pelleted MCs were rapidly frozen at −80 °C. After thawing, aliquots of 50 μL of 4-methyl umbelliferyl-N-acetyl-beta-D-glucosaminide (Sigma-Aldrich, Munich, Germany) solution at 5 μM in citrate buffer (pH 4.5) were mixed with the same volume of supernatant or lysate and incubated for 60 min at 37 °C. The reaction was stopped by 100 mM of sodium carbonate buffer (pH 10.7). Fluorescence intensity was measured at an emission wavelength of 460 nm after excitation at 355 nm. Percent β-hexosaminidase release = [fluorescence intensity SN/(fluorescence intensity SN + fluorescence intensity lysate)] × 100. The net release was calculated by subtracting spontaneous release, as in the histamine release assay above.

### 2.5. Immunoblotting

Detection of ERK1/2, AKT and p38 phosphorylation was as described [33,36]. In brief, MCs were growth factor-deprived and pretreated with or without inhibitors (as specified above) then stimulated at 5 × 10^5^/mL in serum-free medium with AER-37 (0.1 µg/mL) for 30 min, c48/80 (10 µg/mL) or SP (30 µM) for 1 min, or kept without stimulus (at 5 × 10^5^/mL in serum-free medium). Cells were then centrifuged and boiled in SDS-PAGE sample buffer for 10 min. Cell lysates were separated through 4–12% Bis-Tris gels (Thermo Fisher Scientific, Berlin, Germany), then transferred to a blotting membrane and incubation with antibodies. Proteins were visualized by a chemiluminescence assay (Weststar Ultra 2.0, Cyanagen, Bologna, Italy) and bands recorded on a chemiluminescence imager (Fusion FX7 Spectra, Vilber Lourmat, Eberhardzell, Germany). For skin MCs ex vivo, the same protocol was used except for the growth factor-deprivation step.

The following primary antibodies were employed, all purchased from Cell Signaling Technology (Frankfurt am Main, Germany): anti-pp38 (1:1000 dilution, Thr180/Tyr182, #9211), anti-p38 (1:1000 dilution, #9212), anti-pAKT (1:500 dilution, S473, #9271), anti-AKT (1:1000 dilution, #9272), anti-pERK1/2 (1:1000 dilution, T202/Y204, #9101), anti-ERK1/2 (1:1000 dilution, #9102), anti-cyclophilin B (1:25,000 dilution, #43603) and anti-actinin (1:1000 dilution, #6487). Goat anti-rabbit IgG peroxidase-conjugated antibody was administered as the secondary detection antibody (1:10,000 dilution, Merck, #AP132P). The quantification of recorded signals was performed using the ImageJ software (Rasband, W.S., ImageJ, U. S. National Institutes of Health, Bethesda, Maryland, USA, https://imagej.nih.gov/ij/, 1997–2018). Individual intensity values for the detected proteins were normalized to the intensity of the housekeeping protein cyclophilin B or actinin of the same membrane.

### 2.6. Statistical Analysis

Statistical analyses were performed using PRISM 8 (GraphPad Software, La Jolla, CA, USA). RM one-way ANOVA with Dunnett’s multiple comparisons test was performed when data were normally distributed. Friedman’s test with Dunn’s multiple comparisons test was applied when data were not normally distributed. A *p* < 0.05 was considered statistically significant.

The % inhibition was calculated as (1-net release with inhibitor/net release without inhibitor) × 100. The degree of inhibition was compared between MRGPRX2 ligands (c48/80 versus SP) as well as between MC subsets (ex vivo versus cultured). The unpaired *t* test was applied when the data were normally distributed, while the Mann–Whitney U test was applied when the data were not normally distributed.

## 3. Results

### 3.1. G_αi_, G_αq_ and IP3R Channels Contribute to Skin MC Degranulation Triggered via MRGPRX2

**Figure 1:**
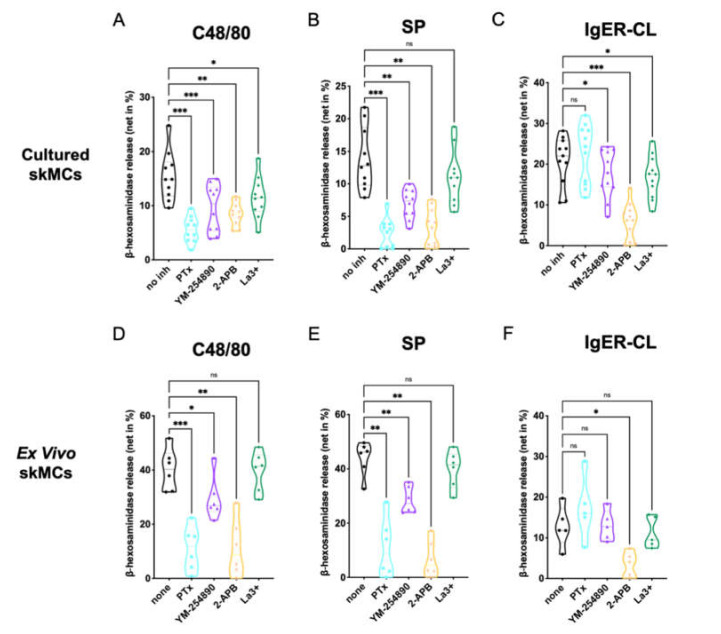
MC degranulation via MRGPRX2 relies on G_αi_, G_αq_ and calcium mobilization. (**A**–**C**) Cultured or (**D**–**F**) ex vivo skin-derived MCs were pretreated with 200 ng/mL PTX, 10 µM YM-254890, 100 µM 2-APB or 1 µM La3+. Then cells were triggered by 10 µg/mL c48/80, 30 µM SP or IgER-CL (cross-linking) (0.1 µg/mL anti-FcεRI-antibody AER-37), β-hexosaminidase release was determined as described in methods. Data are from 5–11 independent experiments, ns: not significant, * *p* < 0.05, ** *p* < 0.01, *** *p* < 0.001, skMCs: skin mast cells.

We analyzed the effects of the G protein inhibitors PTX and YM-254890, as well as the Ca++ channel blockers 2-APB and La3+ on degranulation of skin-derived MCs. Using c48/80 (exogeneous ligand) and SP (endogenous ligand) as MRGPRX2 agonists, we found that both PTX and YM-254890 strongly inhibited β-hexosaminidase release (Figure 1A,B), substantiating the coupling of MRGPRX2 to both G_αi_ and G_αq_. While the combined inositol 1,4,5-trisphosphate/store-operated calcium entry (IP3R/SOCE) blocker 2-APB showed potent suppression [37], the impact of La3+ was generally weaker, yet observable. La3+ inhibits several Ca++ channels, but in MCs, SOCE via Orai1 is likely the most important target [38,39].

No cytotoxicity of the agents was detected at the concentrations and preincubation times used for the experiments (Supplementary Figure S1)

The IgER route was studied for comparison. The most potent effect was found for 2-APB, while weaker suppression by La3+ was also observed (Figure 1C). The G protein inhibitors had no (PTX), or a slight, yet evident effect (YM-254890).

We conclude that in precultured skin MCs, MRGPRX2 requires both G_αi_ and G_αq_ for efficient signaling, and that Ca++ release from *intracellular* stores activated by IP3R on the endoplasmic reticulum (ER) membrane is a crucial element preceding degranulation.

Similar results were found when histamine was measured instead of β-hexosaminidase, substantiating that degranulation of the two preformed mediators depends on the same prerequisites (Supplementary Figure S2A–C).

Next, we studied degranulation responses of ex vivo skin MCs, since differences between freshly extracted and cultured skin MCs have been documented; that can be insignificant or pronounced depending on the gene, protein or process examined [30,40,41,42,43]. For example, MRGPRX2 expression and functional effectiveness are more pronounced in ex vivo MCs, while FcεRI experiences a boost following culture [41]. Again, the inhibitors under the conditions used for experiments had no cytotoxic effects on ex vivo MCs compared to the respective controls (Supplementary Figure S3)

We found that at the level of MRGPRX2-triggered degranulation, both PTX and 2-APB were equipotent in ex vivo MCs, leading to almost complete inhibition (Figure 1D–F). YM-254890 was likewise inhibitory, yet substantially less than PTX. Finally, no significant effect was noted for La3+. The data were thus similar to the findings in precultured MCs, but differences between the G proteins were more accentuated. Concordant findings were also found regarding FcεRI-elicited secretion, in which 2-APB was most suppressive.

To visualize potential differences between c48/80 and SP in Figure 1, we compared suppression between the two ligands side-by-side (Supplementary Figure S4). While the G protein inhibitors displayed comparable inhibition (Supplementary Figure S4A,B,E,F), 2-APB was more potent when SP was used as the MRGPRX2 agonist in cultured, yet not in ex vivo MCs (Supplementary Figure S4C,G). No significant difference was found for La3+ (Supplementary Figure S4D,H).

We also noted that, while inhibition by the distinct inhibitors was comparable by quality in the precultured and ex vivo skin MCs, quantitative differences could be discerned between the subsets according to Figure 1. A direct comparison revealed that G protein inhibitors showed similar inhibition in both types of MCs, 2-APB was more potent in ex vivo MCs after c48/80 stimulation (not for SP), while La3+ tended towards greater inhibition in the precultured subset, yet only reaching significance for c48/80 (Supplementary Figure S5A–H). Regarding FcεRI-triggered secretion, PTX showed no inhibitory, but rather a stimulatory effect, and this was more noticeable in the precultured subset; no differences between cultured and ex vivo MCs were apparent for YM-254890, 2-APB or La3+ (Supplementary Figure S5I–L).

Collectively, crucial signaling events underlying MRGPRX2- and FcεRI-triggered degranulation are generally shared between ex vivo and cultured skin MCs, but some quantitative differences can be observed.

### 3.2. Both ERK and PI3K Contribute to the Degranulation of Skin MCs Stimulated by FcεRI or MRGPRX2

Several kinase activities are involved in FcεRI-dependent MC degranulation (in the introduction), while very little is known for the process in human skin MCs. Moreover, the molecular underpinnings underlying MRGPRX2-elicited degranulation are completely undefined in this physiological MC subset.

**Figure 2:**
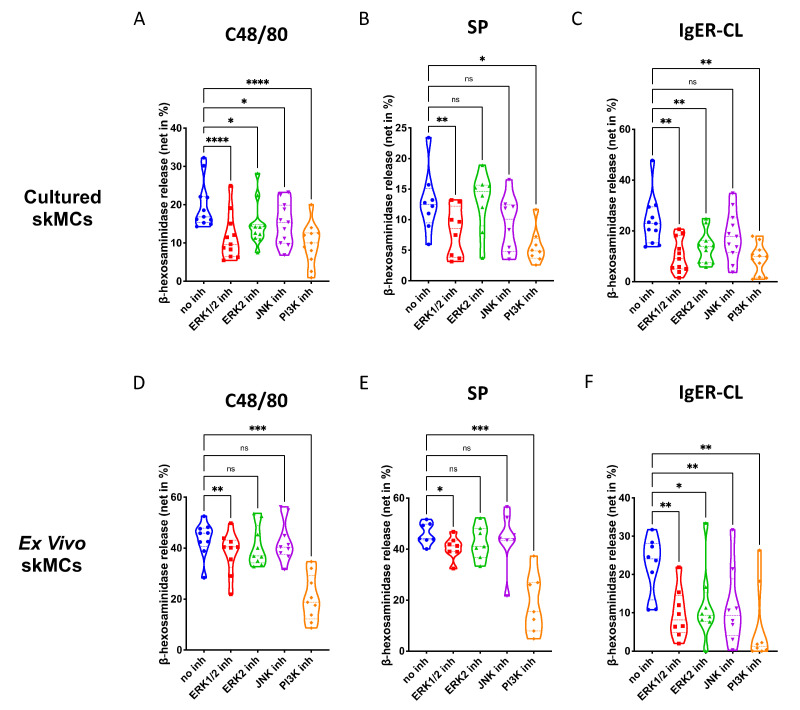
Both ERK and PI3K contribute to the degranulation of skin MCs stimulated by MRGPRX2 or FcεRI. (**A**–**C**) Cultured and (**D**–**F**) ex vivo skin MCs were pretreated with inhibitors targeting ERK1 and ERK2 indiscriminately (SCH772984, 10 µM), ERK2 only (Vx-11e, 2 µM), JNK (SP600125, 5 µM), or PI3K (Pictilisib, 5 µM). β-hexosaminidase release stimulated by c48/80 (10 µg/mL), SP (30 µM) or IgER-cross-linking (CL) (by AER-37 antibody for cultured MCs at 0.1 µg/mL; by anti-FcεRIα-Ab 29C6 for ex vivo MCs at 0.5 µg/mL) was determined. The data are from 7–11 independent experiments, ns: not significant, * *p* < 0.05, ** *p* < 0.01, *** *p* < 0.001, **** *p* < 0.0001. skMCs: skin mast cells.

In skin-derived precultured MCs, we found that ERK1/2 as well as PI3K, significantly contribute to degranulation elicited by the MRGPRX2 agonists 48/80 and SP (Figure 2A,B). The ERK2 inhibitor was less potent, suggesting that either ERK1 or the combination of ERK1 and ERK2 were necessary. The JNK inhibitor was only slightly inhibitory, largely in accordance with our previous work [44]. Moreso, p38 was not included, since we found previously that p38 is not involved in skin MC degranulation (and only becomes a player on potent activation by IL-33) [30]. Exocytosis triggered via FcεRI was susceptible to the same compounds as the MRGPRX2-triggered route, and on average, suppression seemed more pronounced than for MRGPRX2 agonists (Figure 2A–C). Interestingly, the ERK2-selective inhibitor was nearly as suppressive as the one affecting ERK1 and ERK2 indiscriminately, suggesting a greater role of ERK2 in the canonical pathway. Again, all inhibitors employed in this part had no cytotoxic effects in comparison with their respective vehicle controls (Supplementary Figure S6).

The same was true for ex vivo skin MCs (Supplementary Figure S7). As for G proteins and Ca++ channels, the pattern of inhibition was similar by quality to that of precultured equivalents, yet several quantitative differences were detected (Figure 2D–F). Inspecting the two MRGPRX2 agonists side-by-side revealed comparable suppression patterns for c48/80 and SP in both subsets (Supplementary Figure S8). Conversely, when comparing the cell subsets with each other, differences were noted (Supplementary Figure S9). In particular, perturbation of ERK1/2 had a greater impact on MRGPRX2-triggered degranulation in precultured cells, while this was less clear for ERK2 inhibition (the difference was only visible on stimulation with c48/80, but not SP) (Supplementary Figure S9A,B,E,F). The same trend was found for JNK inhibition, i.e., a more pronounced effect in cultured MCs (Supplementary Figure S9C,G). There was no difference detected for PI3K, suggesting that PI3K activity is vital to both subsets, while ERK is likewise important, but in a graduated manner. Interestingly, in the FcεRI-mediated route, no differences were found for ERK inhibition, thus, solidifying the significance of ERK for allergic degranulation of skin MCs irrespective of the environment (Supplementary Figure S9D,H). Only JNK was of greater significance in freshly extracted MCs, while the impact of PI3K was again comparable (Supplementary Figure S9D,H). Collectively, PI3K followed by ERK1/2 crucially contribute to the granule discharge activated by MRGPRX2 agonists. The FcεRI-triggered process is overall more sensitive to the inhibition of key kinases, especially in ex vivo skin MCs.

### 3.3. ERK Activation Depends on G_αi_, G_αq_ and Is Also Inhibited by 2-APB, While AKT Is Selectively Stimulated by G_αi_ upon MRGPRX2 Triggering

We recently reported on strong ERK and detectable but weaker AKT phosphorylation upon MRGPRX2 ligand binding in skin MCs, whereby cytokine stimulation required pERK in the first place [36]. The activation of p38 was weaker, but detectable, while pJNK could not be unambiguously detected [36].

Having determined that MRGPRX2-triggered degranulation depends on G_αi_, G_αq_, Ca++ channels, ERK and PI3K, we set out to investigate the connections between early and later events, also including pp38 for comparison. Blots were repeated 10 or more times to get a solid basis for statistical analyses, allowing for the identification of unifying themes across MC cultures, each containing cells of different (epi-)genomes. In preliminary experiments, we found that La3+ had no effect on any kinase (data not shown) and it was therefore omitted from further analysis.

**Figure 3:**
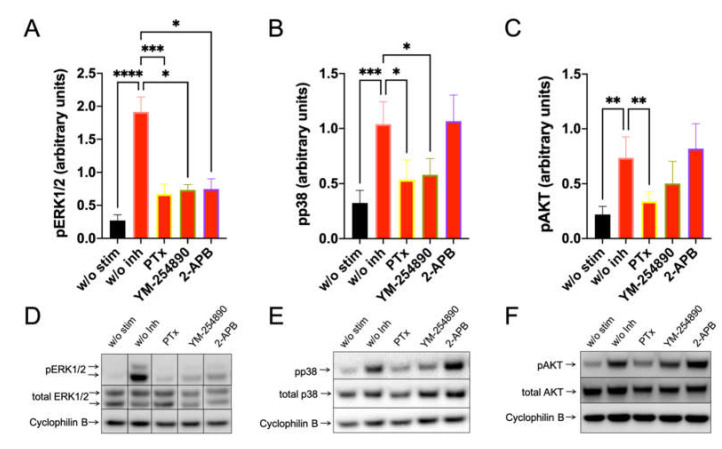
C48/80 activated pERK depends on G_αi_, G_αq_, and Ca++, while pAKT exclusively requires the action of G_αi_ only. Cultured skin-derived MCs were pretreated with PTX (200 ng/mL), YM-254890 (10 µM), 2-APB (100 µM), or no inhibitor (w/o Inh) then stimulated with c48/80 (10 µg/mL) for 1 min. Cells receiving no inhibitor or stimulus were the negative control (w/o stim). (**A**–**C**) Phosphorylation signals detected consecutively on the same membranes for ERK1/2, p38 and AKT, quantified and normalized to cyclophilin B as described in methods. Mean ± SEM of 10–15 independent experiments (individual cultures). * *p* < 0.05, ** *p* < 0.01, *** *p* < 0.001, **** *p* < 0.0001. (**D**–**F**) Representative blots of A–C.

Furthermore, c48/80-induced a very potent pERK1/2 response, which depended on G_αi_, G_αq_ and, unexpectedly, also on 2-APB inhibitable channels (Figure 3, left). Moreso, pp38 showed a similar profile, though no inhibition by 2-APB was observed (Figure 3, center). Most interestingly, however, was that pAKT was exclusively activated by G_αi_, but not by G_αq_, since YM-254890 had no effect (Figure 3, right). Additionally, 2-APB was likewise ineffective at the level of pAKT. Similar results were obtained by normalizing against the alternative housekeeping gene actinin (Supplementary Figure S10).

**Figure 4:**
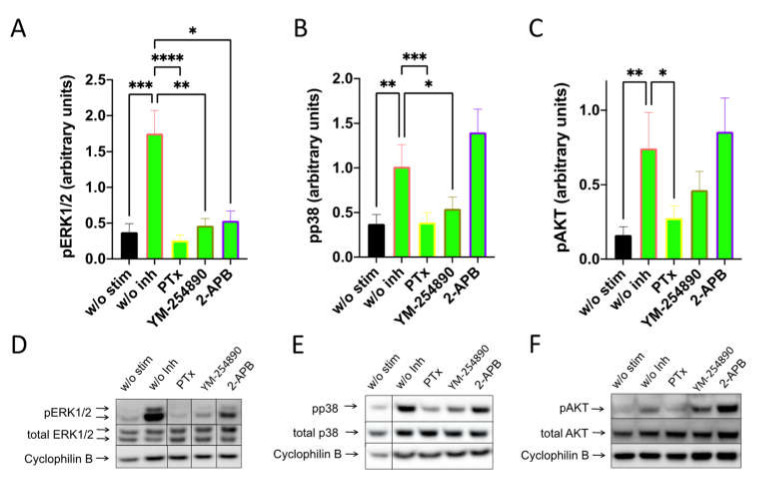
Interconnections between early and later signals induced by SP are comparable to those elicited by c48/80. MCs were pretreated with inhibitors, as shown in Figure 3, then stimulated with SP (30 µM) for 1 min. (**A**–**C**) Signals of pERK1/2, pp38 and pAKT were quantified and normalized to cyclophilin B as described in methods. (**D**–**F**) Representative blots of A–C. Data shown are Mean ± SEM of 11–13 independent experiments. * *p* < 0.05, ** *p* < 0.01, *** *p* < 0.001, **** *p* < 0.0001.

By investigating SP-stimulated kinases, we found a comparable profile: pERK1/2 was suppressed to a similar degree by the three inhibitors. Furthermore, G_αi_ and G_αq_ were both linked to p38 activation, while the appearance of pAKT was exclusively countered by PTX (Figure 4). Quantification against actinin gave comparable results and can be found in Supplementary Figure S11.

We recently found that kinase activation kinetics were largely equal in cultured and ex vivo MCs [36]. Here, we assessed the impact of inhibitors on MRGPRX2-triggered kinase stimulation in freshly extracted MCs. This experiment could be performed three times. As in cultured MCs, PTX interfered with the phosphorylation of both pERK1/2 and pAKT upon c48/80 and SP stimulation also in ex vivo MCs (Supplementary Figure S12A–D). For YM-254890 and 2-APB, a tendency was noted on c48/80 stimulation (Supplementary Figure S12A). Furthermore, pp38 was barely induced in ex vivo MCs because of high baseline phosphorylation (not shown).

Collectively, especially G_αi_ is involved in the orchestration of key signaling molecules downstream of MRGPRX2, while G_αq_ and Ca++ channels can contribute to ERK activation.

### 3.4. Influence of G Protein and Calcium Channel Inhibitors on Kinase Activation via FcεRI in Skin MCs

**Figure 5:**
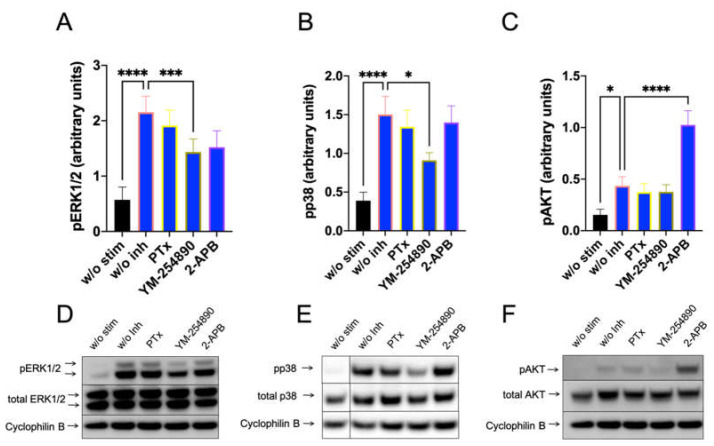
Ca++ channel inhibition boosts AKT activation downstream of IgER-CL. MCs were pretreated with inhibitors, as described in Figure 3, then stimulated for 30 min with AER-37 (0.1 µg/mL) for FcεRI-aggregation. (**A**–**C**) Signals of pERK1/2, pp38 and pAKT were quantified and normalized to cyclophilin B as described in methods. (**D**–**F**) Representative blots of A–C. *n* = 10–13, Mean ± SEM. * *p* < 0.05, *** *p* < 0.001, **** *p* < 0.0001.

We studied whether G_αi_, G_αq_ and Ca++ channels are linked to downstream pathways that are activated by FcεRI-aggregation using precultured skin MCs, in which FcεRI can be more potently activated [41]. As expected, PTX was without effect on pERK, pp38 and pAKT stimulation (Figure 5). Surprisingly, YM-254890 slightly (but significantly) countered the appearance of both pERK and pp38. Interestingly, in contrast to the MRGPRX2 pathway, 2-APB had no effect on pERK stimulated via the IgE-dependent route, indicating a distinct dependence on Ca++ in MRGPRX2 vis-à-vis FcεRI-induced ERK phosphorylation in cultured MCs (Figure 5 versus Figure 3 or Figure 4 , or Supplementary Figure S13 versus Supplementary Figure S10 or S11). Similar results were observed when actinin was used for quantification (Supplementary Figure S13). Even more, 2-APB had a promoting effect on PI3K/AKT, suggesting that 2-APB sensitive channels were negative regulators of pAKT in the IgE-dependent route, but not in the MRGPRX2-driven pathway. Overall, despite the activation of similar kinases by both receptor systems, the prerequisites leading to their activation differ.

## 4. Discussion

Hypersensitivity reactions can be owed to allergic or the clinically indistinguishable pseudo-allergic activation of MCs and depend on the acute release of high amounts of preformed mediators in a process termed degranulation or granule exocytosis [1,3,5,8,45]. As the receptor of pseudo-allergic and neurogenic MC responses, MRGPRX2 has come to the forefront of scientific interest in recent years. Due to its abundant expression in skin MCs [17,18,19,20] and the ever-growing list of ligands [1,3,6,7], MRGPRX2 acts as the key inducer of IgE-independent reactions and greatly expands the functional spectrum of MCs, especially in the cutaneous environment [1,2,46,47], the organ with greatest MC density [16].

Since the recognition of MRGPRX2’s significance in MC biology started recently, signaling events triggered by MRGPRX2 are still rudimentary, and except for one study from our laboratory [36], nothing is known about skin MCs. Our previous report focused on the activation of selected kinases in the context of cytokine generation; an important outcome was the rapidness of MRGPRX2 signaling vis-à-vis FcεRI-aggregation, the latter substantially delayed [36].

The current study explores the mechanistic prerequisites underlying MRGPRX2-elicited degranulation in dermal MCs, i.e., the acute process behind hypersensitivity, urticaria and anaphylaxis. Importantly, the two stimulatory systems, i.e., allergic and pseudo-allergic, are directly compared with each other. Since MRGPRX2 represents a GPCR, we first focused on the G proteins activated downstream in skin MCs. Early work suggested that MRGPRX2 is chiefly G_αq_ coupled, at least in (transfected) HEK293 cells [48]. However, later work found both G_αi_ and G_αq_ involvement. For example, in LAD2 MCs stimulated with host defense peptides (human β-defensin-3, or LL-37, both serving as MRGPRX2 agonists), degranulation was basically abolished by PTX (YM-254890 was not tested) [49,50]. Likewise, PTX almost completely abrogated β-hexosaminidase release stimulated by icatibant (a drug capable of activating MCs via MRGPRX2) in RBL-MRGPRX2 (i.e., RBL-2H3 cells stably transfected with human MRGPRX2) [15]. Later, PTX and YM-254890 were compared side-by-side, both effectively interfering with SP-triggered exocytosis in the same cells [51]. In mastoparan-activated HEK293-MRGPRX2 cells, however, only G_αq_ activation was observed [52]. Using transducerome screening by a technique named Trupath, a recent study found that MRGPRX2 can couple to most of the 14 G_α_ proteins at least under the conditions of overexpression in HEK293 transfectants [53]. The collective data hint at variability across cell types with non-MCs (such as MRGPRX2-transfected HEK293) chiefly employing G_αq_ (in the absence of G protein overexpression), while cells with MC characteristics (LAD2, RBL-MRGPRX2) showed coupling to both G_αi_ and G_αq_. In accordance, we uncover for skin MCs as the physiologically relevant subset that both G_αi_ and G_αq_ contribute significantly to granule discharge. However, in contrast to RBL-MRGPRX2, in which G_αi_ and G_αq_ inhibitors displayed similar levels of inhibition [51], G_αi_ seems to occupy a more significant role in our MC subsets, both precultured and, even more so, in MCs freshly extracted from the skin. Due to cell-to-cell variability, it is therefore essential to clarify the molecular underpinnings of MRGPRX2 signaling in physiological MCs that endogenously express all components in their natural ratios.

Degranulation-competent receptors like FcεRI and MRGPRX2 induce a calcium signal. In contrast, receptors incapable of inducing a Ca++ pulse (e.g., ST2/IL-33R or TSLPR) do not lead to degranulation, even though they can influence or prime the degranulation response induced by other receptors, especially FcεRI and MRGPRX2 [30,31,44]. In the case of MRGPRX2, G protein activation is followed by PLCβ (and/or PLCγ) activation [8,54], enabling Ca++ channels to facilitate its increase in the cytoplasm. This exocytotic prerequisite subsequently enables granule fusion with the plasma membrane through interactions with the degranulation apparatus, including synaptotagmin, and the regulation of actin depolymerization [55,56]. Important Ca++ sources in MCs are the depletion of internal stores (mainly via IP3R on the ER membrane), and entry from the extracellular space by SOCE. The latter pathway chiefly uses the STIM/Orai1 system (and to a lesser extent, channels of the TRP family) [57,58]. Recently, Occhiuto et al. found a crucial role of SOCE for the MRGPRX2-driven route in LAD2 cells [59]. Similar to MCs from lung tissue [57,60], skin MCs express abundant levels of Orai channels (especially ORAI1 and ORAI3) [17]. Moreso, 2-APB interferes with both IP3R and SOCE [61,62], as also summarized in Supplementary Table S1. To the best of our knowledge, the contribution of different sources has not been investigated in the context of skin MC degranulation. Here, we show that 2-APB is highly effective at reducing degranulation by both receptor systems. In this regard, 2-APB is substantially more suppressive than La3+, the latter inhibiting Orai-dependent SOCE, as demonstrated in primary human lung MCs [60]. Since La3+ can only inhibit entry from extracellular sources, while 2-APB also antagonizes IP3R-mobilized Ca++, our results highlight the significance of ER-derived Ca++ beyond its role as a SOCE initiator in both stimulatory pathways. This statement may be more relevant for SP- than for c48/80-induced degranulation, since inhibition by 2-APB was significantly more pronounced after stimulation by SP, at least in cultured MCs. In contrast, in MRGPRX2-mediated degranulation of LAD2 cells, both 2-APB and La3+ comparably attenuated β-hexosaminidase release [49].

Common downstream events shared by most stimulatory receptors (though with distinct efficiencies and/or kinetics) encompass MAPKs (ERK1/2, p38, JNK) and PI3K/AKT. These constituents are also well-documented for the FcεRI-driven route [26,63,64,65,66], but even for the canonical route, evidence is mostly based on murine studies (BMMCs). Far fewer reports are available for MRGPRX2 due to its relative novelty.

For human skin MCs, we recently demonstrated that all the above kinases are activated upon MRGPRX2 ligation, but with variable efficiency in the following order pERK1/2 > pAKT > pp38 > pJNK, whereby JNK modulation was weak and was observed only occasionally [36]. The comparison of time-courses was particularly striking, since MRGPRX2-elicited rapid responses (maximum ~1 min), while modulations by FcεRI were substantially delayed (8–30 min).

Isolated reports on kinase activation and involvement in MRGPRX2-driven exocytosis can be found in the literature [54,67,68,69,70]; in addition to the distinct G protein coupling described above, they, too, indicate differences across MC subsets. For example, Azzolina et al. found swift phosphorylation of p38 and JNK but relatively delayed ERK1/2 phosphorylation in rat peritoneal MCs after SP stimulation [67]. Conversely, codeine activation of LAD2 cells elicited prompt phosphorylation of ERK and JNK but not of p38 [71].

Here, we confirm rapid phosphorylation of ERK, AKT and, to a lesser degree, p38 and find that both ERK1/2 and PI3K/AKT are required for efficient degranulation of skin MCs. While the involvement of the latter was expected, at least for the well-explored allergic route, ERK1/2 was surprising considering that over decades, ERK was associated with various functions, including cytokine and eicosanoid generation, yet not with granule exocytosis [25,26,27,28]. However, in accordance with our study, a recent report pinpointed ERK as a key player in MC degranulation stimulated by MRGPRX2 in the MC line LAD2 [54]. The authors reported that the reason for the discrepancy was a major difference between mouse and human MCs since only the latter depended on ERK for MRGPRX2-driven β-hexosaminidase and histamine release [54]. This was a notable finding since MC research is heavily biased towards the mouse, and makes vivid use of murine BMMCs, which differ not only from humans, but also from other murine MC types [72]. In our study, ERK involvement was found for both ex vivo and precultured skin MCs, yet it was stronger in the latter, suggesting that the cellular environment also plays a role in dictating the significance of ERK. We moreover reveal that the FcεRI-elicited response (not covered by the recent study) likewise relies on ERK in human (skin) MCs. Viewed against the literature [25,26,27,28], this result likewise suggests major differences in FcεRI-driven degranulation between human and rodent MCs (BMMCs in particular). While the distinct levels at which ERK operates to facilitate MC degranulation will require future efforts (ERK has more than 100 substrates [73]), a previous study reported that at least under certain conditions, optimal activation of PLCγ1 can require ERK1/2 [69]. For FcεRI, no difference was noted regarding ERK involvement between freshly extracted and cultured MCs, implying a crucial role for ERK in the granule discharge of skin MC in response to allergic stimuli under all circumstances. Collectively, evidence is accumulating that different MCs use distinct components of their signaling machinery for the same functional outputs, such as degranulation, the most MC-selective and acutely harmful process.

Frequently, kinase activation and upstream events are studied in isolation without the attempt to interconnect the different levels. Here, we were interested in understanding whether and how early signaling events like G proteins and Ca++ channels are intersected with the activation of kinases further downstream.

We found that pERK activation via MRGPRX2 depends on multiple upstream events, i.e., G_αi_, G_αq_ and 2-APB-inhibitable channels (while being refractory to La3+). Moreso, p38 activation required G_αi_ and G_αq_ and, most interestingly, pAKT only depended on G_αi_. G_αi_ was also the most remarkable partaker in ex vivo MCs. That AKT phosphorylation is selectively inhibited by PTX may explain the greater impact of PTX versus YM-254890 on degranulation, emphasizing that in skin MCs, G_αi_ may be more relevant than G_αq_ to MRGPRX2-triggered biological outcomes. This is further corroborated by the significance of both G_αi_ and PI3K in freshly extracted MCs. On the other hand, the observation that 2-APB can inhibit ERK phosphorylation indicated that IP3R channels are involved (note that La3+ interfering with SOCE had no effect) at least in precultured MCs. This suppressive effect was unexpected, since the involvement of Ca++ in the activation of ERK is rather uncommon, even though it has been reported. For example, inhibition of calmodulin or Ca++ channels interfered with ERK phosphorylation in an astrocytoma cell line [74]. In the transactivation of EGFR, 2-APB likewise prevented ERK activation [75], as it did in neutrophils stimulated by PAF [76]. In one study, Ca++ was shown to activate ERK in MCs [39]. The effects are cell-dependent, because, in the differently activated endothelial cells, 2-APB did not modulate ERK activity (while inhibiting pAKT) [77,78]. In contrast, we did not observe inhibition of pAKT by 2-APB.

Intriguingly, despite similar kinase activation patterns by MRGPRX2 and FcεRI, the in-depth examination of how activities are brought about unearths inter-route differences, though FcεRI was not the focus and only included for comparative reasons. We certainly did not expect to find the effects of G protein inhibitors. In accordance, PTX had no effect on degranulation or signaling following FcεRI-aggregation, while PTX was the most effective inhibitor for the MRGPRX2 route, blunting phosphorylation of all examined kinases. However, YM-254890 not only interfered with pERK and pp38 after stimulation with SP or c48/80, but it was also effective at suppressing the same kinase activities in the FcεRI pathway, at least to a modest degree. This suggests either the necessity of cross-talk between GPCRs (not necessarily MRGPRX2) and FcεRI or an unappreciated involvement of G_αq_ in IgE-dependent stimulation. In fact, other GPCRs, e.g., sphingosine-1-phosphate receptor 2, can dictate the effectiveness of FcεRI-triggered degranulation [79]. Moreover, it was also reported that β-arrestin-2 (activated downstream of GPCRs to orchestrate GPCR desensitization) not only interferes with MRGPRX2-triggered degranulation but is also a negative regulator of the FcεRI-triggered process in the mouse [80]. The enhanced responsiveness to Ca++ ionophore in β-arrestin-2 deficient MCs indicated that β-arrestin-2 is rather a general regulator of MC function in this latter case [80]. Clearly, more research will be needed to delineate how these interconnections are molecularly governed and whether they depend on the precise MC subset.

While no effects of G protein inhibitors were expected for the pathways elicited by FcεRI, Ca++ is an essential component of both receptor systems. Here, it was particularly surprising that 2-APB can enhance AKT phosphorylation. To the best of our knowledge, such a phenomenon has not been described. On the contrary, in neutrophils stimulated by PAF, 2-APB suppressed PI3K activation [76]. Similarly, in human endothelial cells, 2-APB selectively inhibited VEGF- or angiopoietin-induced activation of PI3K/AKT [77,78] as well as in BAFF-stimulated B cells [81]. Our result, therefore, suggests an MC-selective and even receptor-dependent phenomenon, since it occurred only upon FcεRI cross-linking. It may be an inherent counter-regulatory mechanism built into the pathway to offset overpowering degranulation, that can be tissue-destructive. It could be related to the finding that, under certain circumstances, PI3K can be suppressed by (Ca++ dependent) PKC as an intermediary [82]. Its confinement to MCs may be explained by the uniqueness of MCs being well-separated from other hematopoietic and non-hematopoietic constituents [16,17,20,83,84,85]. The observation, as such, is intriguing, and its molecular details will deserve future research.

It is also of interest that 2-APB inhibited ERK activation upon MRGPRX2 ligand binding, as least in cultured MCs, but was without such effect in the FcεRI-driven route, even though 2-APB had similar consequences on degranulation stimulated by both receptors. This emphasizes that the signaling cascades that lead up to the activation of MAPKs and AKT differ between allergic and pseudo-allergic/neurogenic activation, even if they superficially resemble each other.

In summary, our study shows that MRGPRX2-driven degranulation of skin MCs requires the action of G_αi_, G_αq_, Ca++ channels (including IP3Rs), PI3K and ERK. G proteins trigger downstream kinases, of which two are crucial to organize granule exocytosis in skin MCs, namely PI3K (expected, dominant) and ERK (unexpected, less pronounced). The intracellular events leading to kinase activation in the receptor systems differ, as best exemplified by the differential effects of 2-APB, which suppresses degranulation similarly in the two systems, yet probably through distinct downstream effectors. Our study is an important contribution to understanding signaling networks underlying granule discharge of primary human MCs.

## 5. Conclusions

Not only PI3K, but to some degree also ERK1/2 contribute to MRGPRX2- and FcεRI-driven degranulation in primary human skin MCs, assigning a broader role to ERK than hitherto suspected. Granule discharge by MRGPRX2 also depends on G_αi_, G_αq_, and Ca++ channels. While ERK activation can require several parallel upstream events, pAKT appearance depends exclusively on G_αi_. G_αi_ is, overall, the most important G protein activated by MRGPRX2 in skin MCs. Overlapping kinases are activated downstream of FcεRI and MRGPRX2, but upstream events differ between the receptors. Strikingly, interference with IP3R-derived Ca++ boosts pAKT selectively in the FcεRI route. This study crucially provides a first signaling framework underlying the degranulation of skin MCs and reveals unexpected interdependencies at distinct levels of the signal transmission apparatus in these physiological MCs.

## Data Availability

Not applicable.

## Supplementary Materials

The following supporting information can be downloaded at: https://www.mdpi.com/article/10.3390/cells11060953/s1, Figure S1: The inhibitors employed in main Figure 1 exert no cytotoxicity on cultured skin MC; Figure S2: MC degranulation via MRGPRX2 relies on Gαi, Gαq and calcium mobilization as evidenced by histamine release; Figure S3: The survival of ex vivo skin MC is not perceptibly compromised by the inhibitors employed in main Figure 1; Figure S4: Differences between c48/80 and SP from main Figure 1; Figure S5: Comparison between cultured and ex vivo skin MCs from main Figure 1; Figure S6: The inhibitors employed in main Figure 2 exert no cytotoxicity on cultured skin MC; Figure S7: The survival of ex vivo skin MCs is not perceptibly compromised by the inhibitors employed in main Figure 2; Figure S8: Differences between c48/80 and SP from main Figure 2; Figure S9: Comparison between cultured and ex vivo skin MCs from main Figure 2; Figure S10: c48/80 activated pERK depends on Gαi, Gαq, and Ca++, while pAKT exclusively requires the action of Gαi only—normalization against actinin; Figure S11: Interconnections between early and later signals induced by SP are comparable to those elicited by c48/80—normalization against actinin; Figure S12: Interconnections between early and later signals induced by c48/80 and SP in ex vivo MCs; Figure S13: Ca++ channel inhibition boosts AKT activation downstream of IgER-CL—normalization against actinin; Table S1: Inhibitors employed and their primary targets. References [15,30,31,33,36,38,39,44,49,51,55,60,61,62,86,87,88,89,90,91,92,93,94,95,96] are cited in the Supplementary materials.
