# Supplementary material for: MRGPRX2-Mediated Degranulation of Human Skin Mast Cells Requires the Operation of Gαi, Gαq, Ca++ Channels, ERK1/2 and PI3K—Interconnection between Early and Late Signaling
Source: Cells. 2022 Mar 10;11(6):953. doi: 10.3390/cells11060953 (PMC8946553; doi:10.3390/cells11060953)
Supplement: Supplementary file 1 [file cells-11-00953-s001.zip › cells-1578178-SI.pdf]

cells
MDPI

ORIGINAL ARTICLE – ONLINE SUPPLEMENT

MRGPRX2-Mediated Degranulation of Human Skin Mast Cells Requires the Operation of Gαi, Gαq, Ca++ Channels, ERK1/2 and PI3K—Interconnection between Early and Late Signaling

Zhao Wang [1,2,†], Kristin Franke [1,3,†], Gürkan Bal [1,3], Zhuoran Li [1,3], Torsten Zuberbier [1,3] and Magda Babina [1,3,*]

[1] Institute for Allergology, Charité—Universitätsmedizin Berlin, Corporate Member of Freie Universität Berlin, Humboldt-Universität zu Berlin, and Berlin Institute of Health, 10117 Berlin, Germany; wang.zhao@xjtu.edu.cn (Z.W.); kristin.franke@charite.de (K.F.); guerkan.bal@charite.de (G.B.); zhuoran.li@charite.de (Z.L.); torsten.zuberbier@charite.de (T.Z.)
[2] Department of Dermatology, The Second Affiliated Hospital, Northwest Hospital, Xi'an Jiaotong University, Xi'an 710004, China
[3] Fraunhofer Institute for Translational Medicine and Pharmacology ITMP, Allergology and Immunology, 12203 Berlin, Germany
* Correspondence: magda.babina@charite.de
† These authors contributed equally to this work.

Supplementary Table

Supplementary Table S1. Inhibitors employed and their primary targets.

Inhibitor	Function	Supplier
Pertussis toxin	G protein inhibitor [1-3]<br>$G_{\alpha i/o}$ inhibitor [4-8]	List Biological Labs
YM-254890	Selective $G_{\alpha q}$ inhibitor [9-11]	Fujifilm
2-APB	Inositol 1,4,5-trisphosphate receptor inhibitor [8,12,13]<br>Store-operated calcium entry (SOCE) inhibitor [14]<br>Ca++ release activated channels (CRAC) inhibitor [12,14,15]	Santa Cruz Biotechnology
La3+	SOCE inhibitor [16]<br>CRAC blocker [17]	Sigma-Aldrich
SP600125	JNK inhibitor[18-22]	ApexBio
Pictilisib	PI3K inhibitor[18]	Selleckchem
SCH772984	ERK1/2 inhibitor[18]	BioVision
Vx-11e	ERK2 inhibitor[18]	BioVision

Supplementary Figures

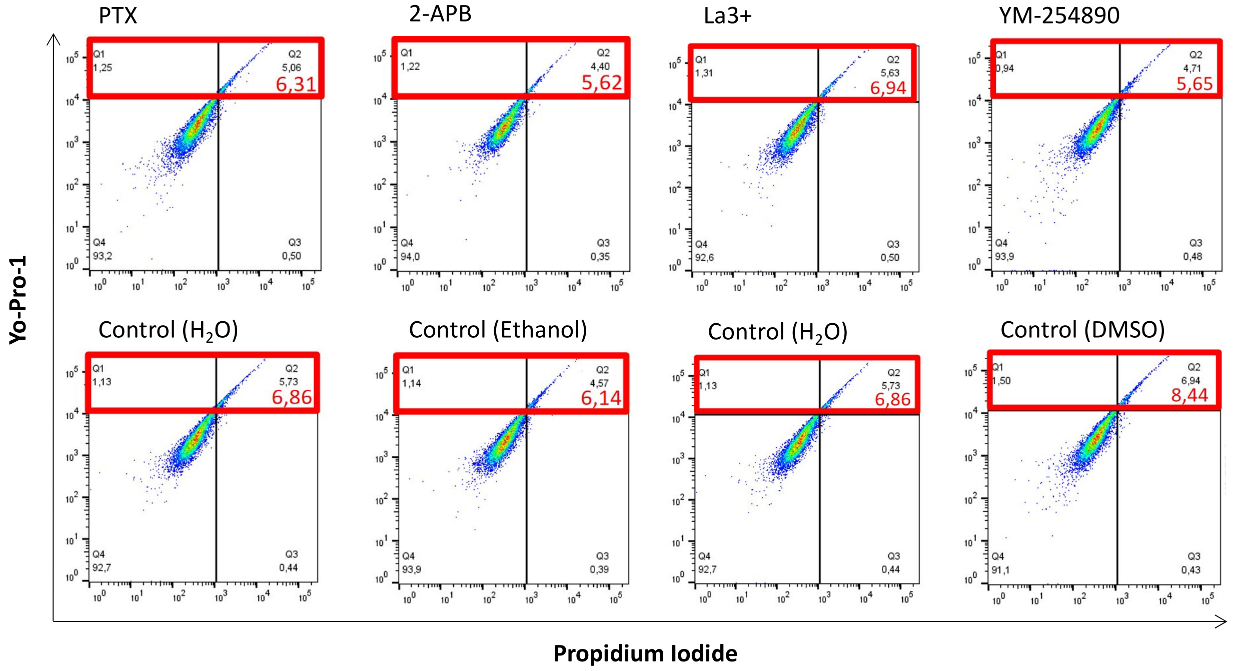

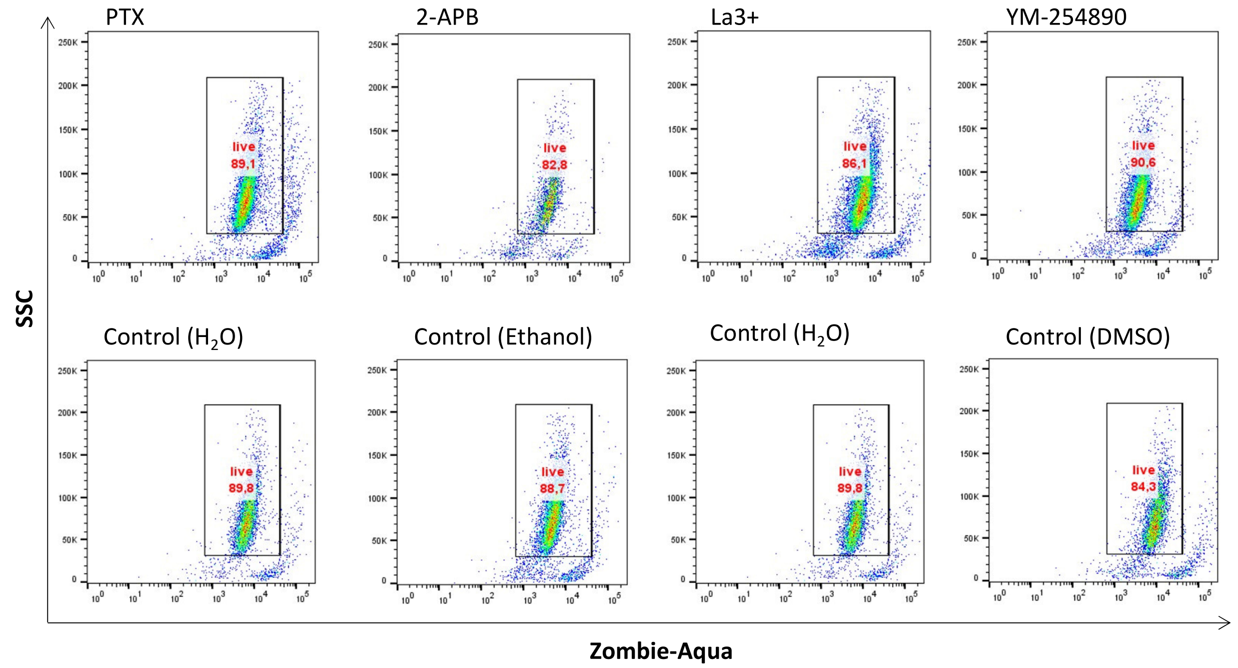

Supplementary Figure S1. The inhibitors employed in main Fig. 1 exert no cytotoxicity on cultured skin MC. Cultured skin-derived MCs were incubated with PTX, 2-APB, YM-254890, La3+, or with corresponding controls (solvent of each inhibitor) at the concentrations and preincubation times used for experiments and described in Methods. Each control is given below the respective inhibitor. YO-PRO-1/Propidium Iodide were stained to detect apoptosis, Zombie Aqua was used to quantify viability. The red frames indicate the total percentage of apoptotic cells given in as red numbers. Cells were measured by flow cytometry. 1 out of 2 experiments is shown. Note that viability is high and not compromised by any inhibitor.

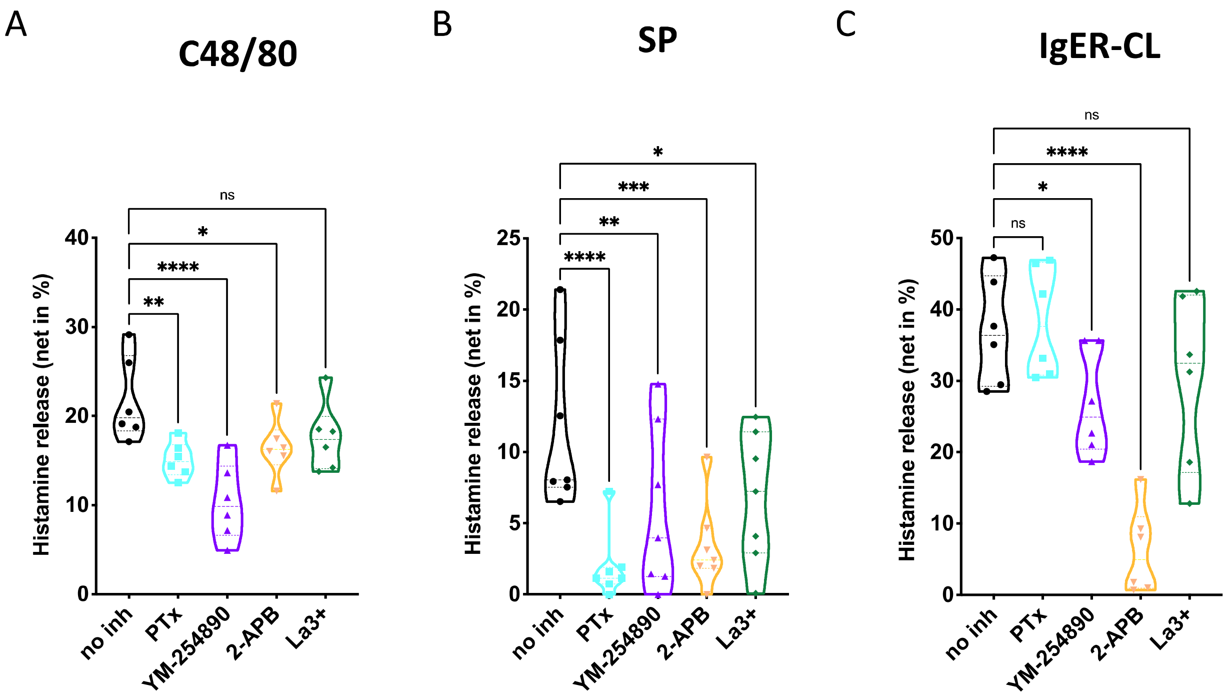

Supplementary Figure S2. MC degranulation via MRGPRX2 relies on Gαi, Gαq and calcium mobilization as evidenced by histamine release. Cultured skin derived MCs were pre-treated with PTX, YM-254890, 2-APB or La3+, then triggered by (A) c48/80, (B) SP or (C) IgER-CL (cross-linking) as in main Fig. 1, histamine release was determined. The data are from 6-7 independent experiments, ns: not significant, * $p < 0.05$, ** $p < 0.01$, *** $p < 0.001$, **** $p < 0.0001$.

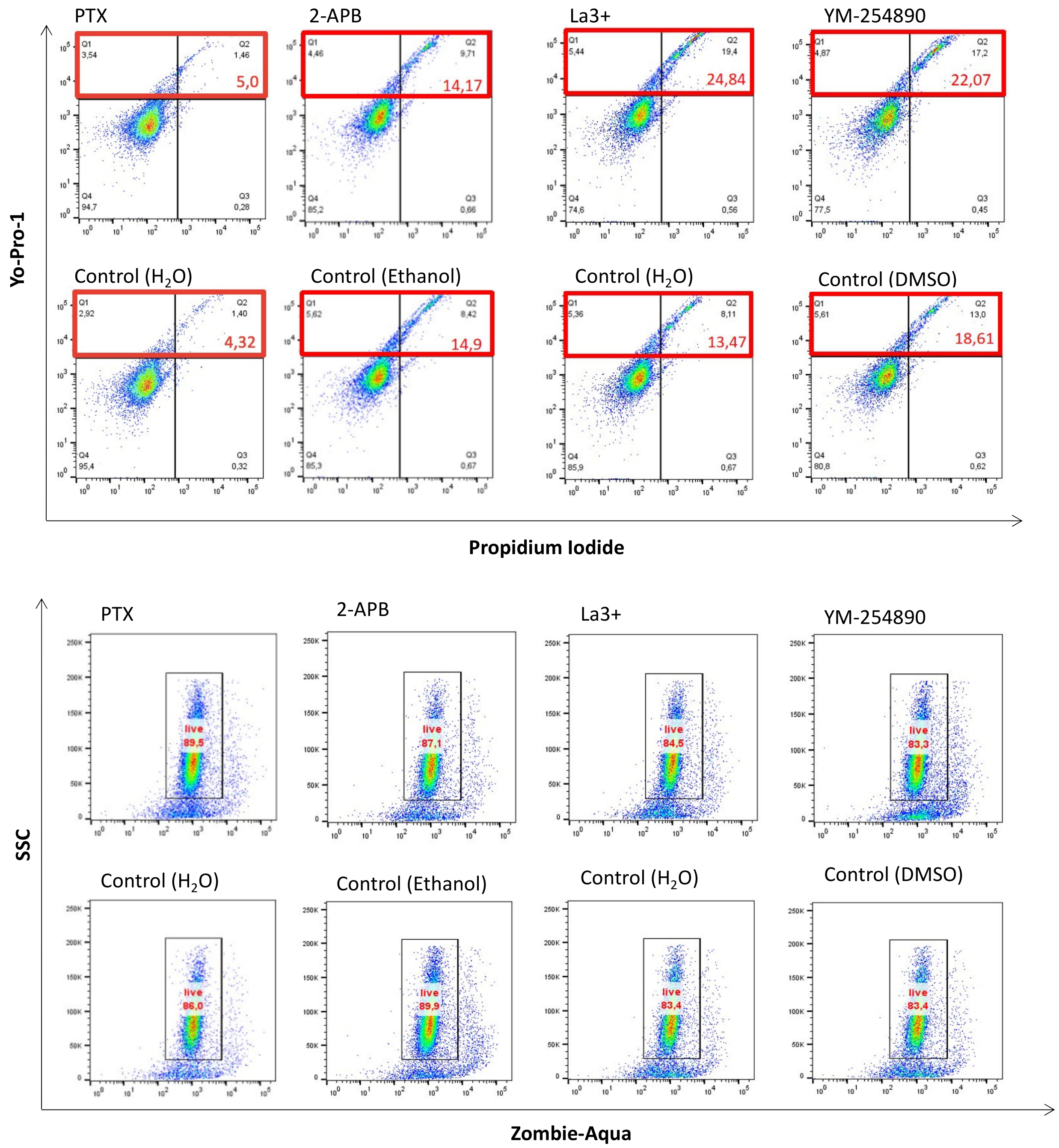

Supplementary Figure S3. The survival of *ex vivo* skin MC is not perceptibly compromised by the inhibitors employed in main Fig. 1. *Ex vivo* skin MCs were incubated with PTX, 2-APB, YM-254890, La3+, or with corresponding controls (solvent of each inhibitor) at the concentrations and preincubation times used for experiments and described in Methods. Each control is given below the respective inhibitor. YO-PRO-1/Propidium Iodide were stained to detect apoptosis, Zombie Aqua was used to quantify viability. The red frames indicate the total percentage of apoptotic cells given in as red numbers. Cells were measured by flow cytometry. Results are pooled from 2 different experiments. Note that viability is high and not compromised by any inhibitor.

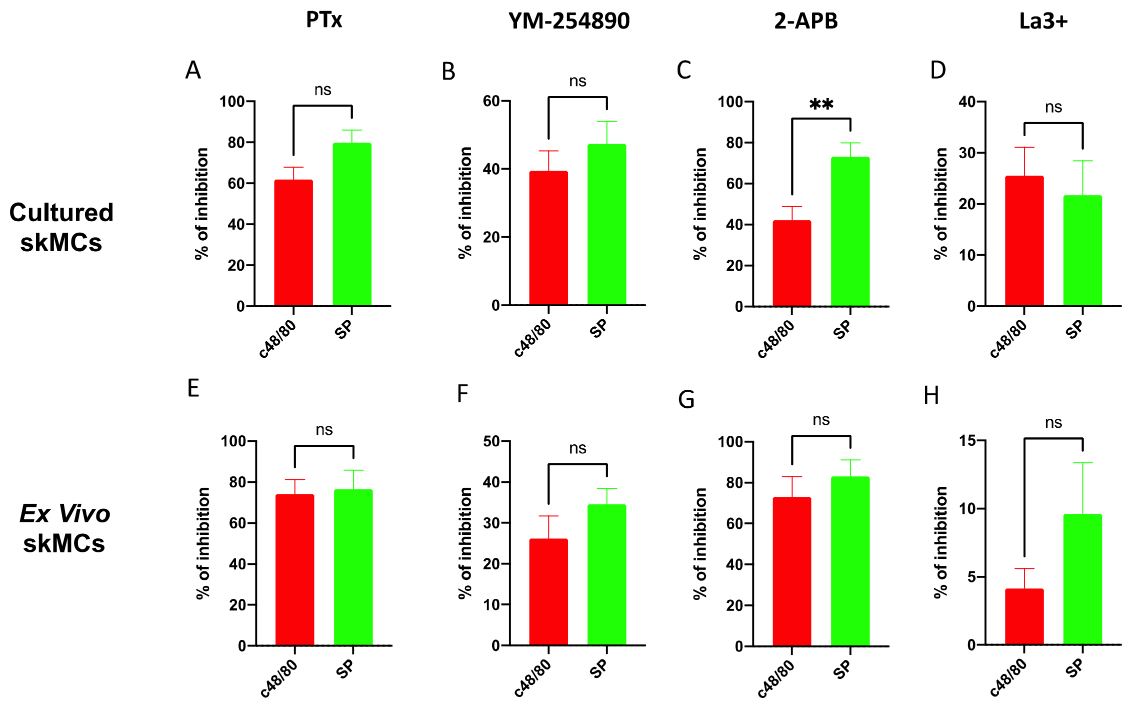

Supplementary Figure S4. Differences between c48/80 and SP from main Fig. 1. (A-D) Cultured or (E-H) *ex vivo* skin derived MCs were pre-treated with PTX, YM-254890, 2-APB or La3+ and triggered by c48/80 or SP, β-hexosaminidase release was measured according to Fig 1. % inhibition (calculated as described in Methods) was compared between c48/80 and SP. The data are from 6-10 independent experiments, ns: not significant, ** $p < 0.01$, skMCs: skin mast cells.

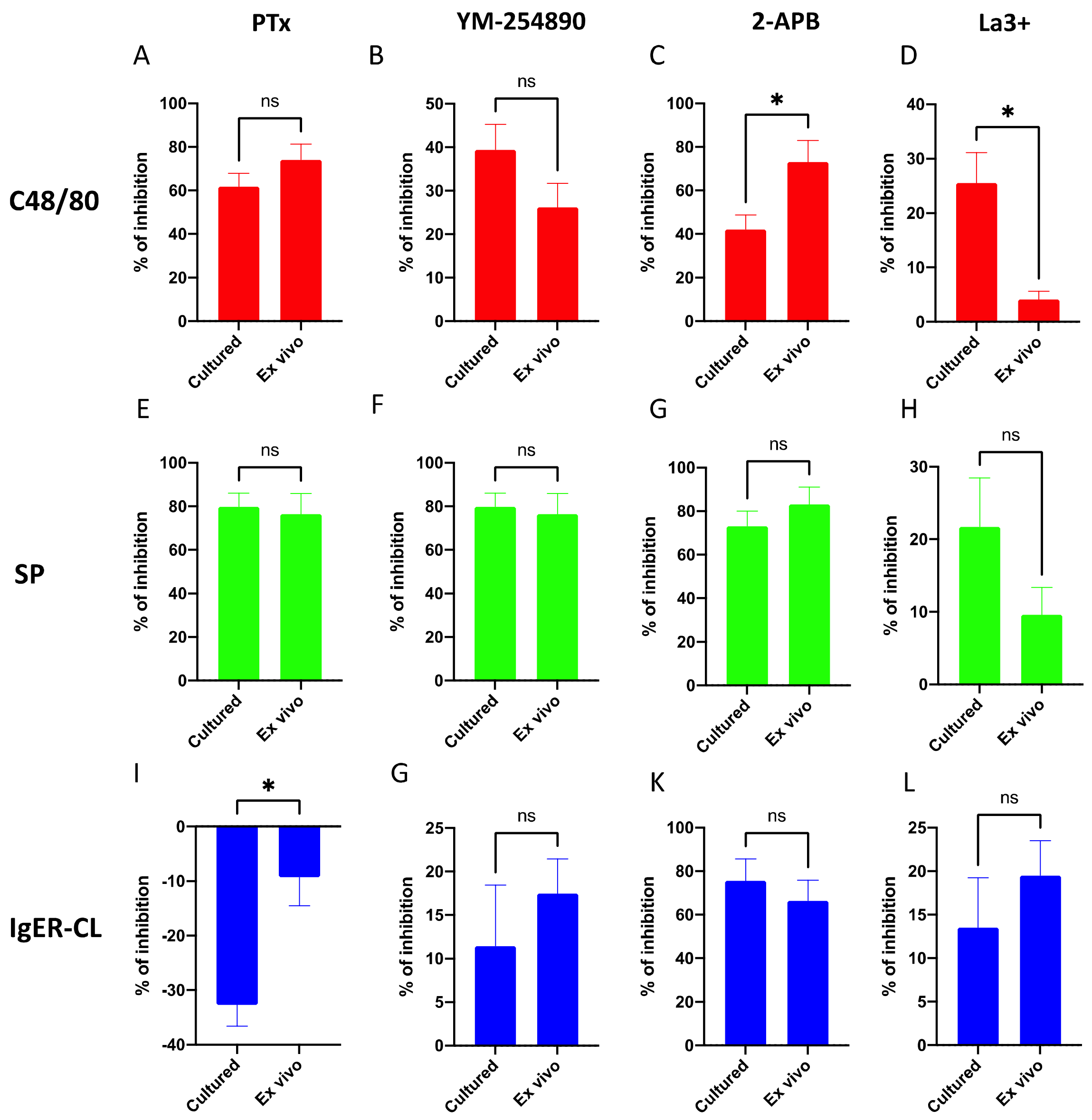

Supplementary Figure S5. Comparison between cultured and *ex vivo* skin MCs from main Fig. 1. Cultured and *ex vivo* skin MCs were pre-treated with PTX, YM-254890, 2-APB or La3+ then stimulated by c48/80, SP or IgER-CL (cross-linking) according to Fig 1. β-hexosaminidase release was determined. % inhibition (calculated as described in Methods) for (A-D) c48/80, (E-H) SP or (I-L) IgER-CL was compared between the MC subsets. The data are from 5-12 independent experiments, ns: not significant, * $p < 0.05$.

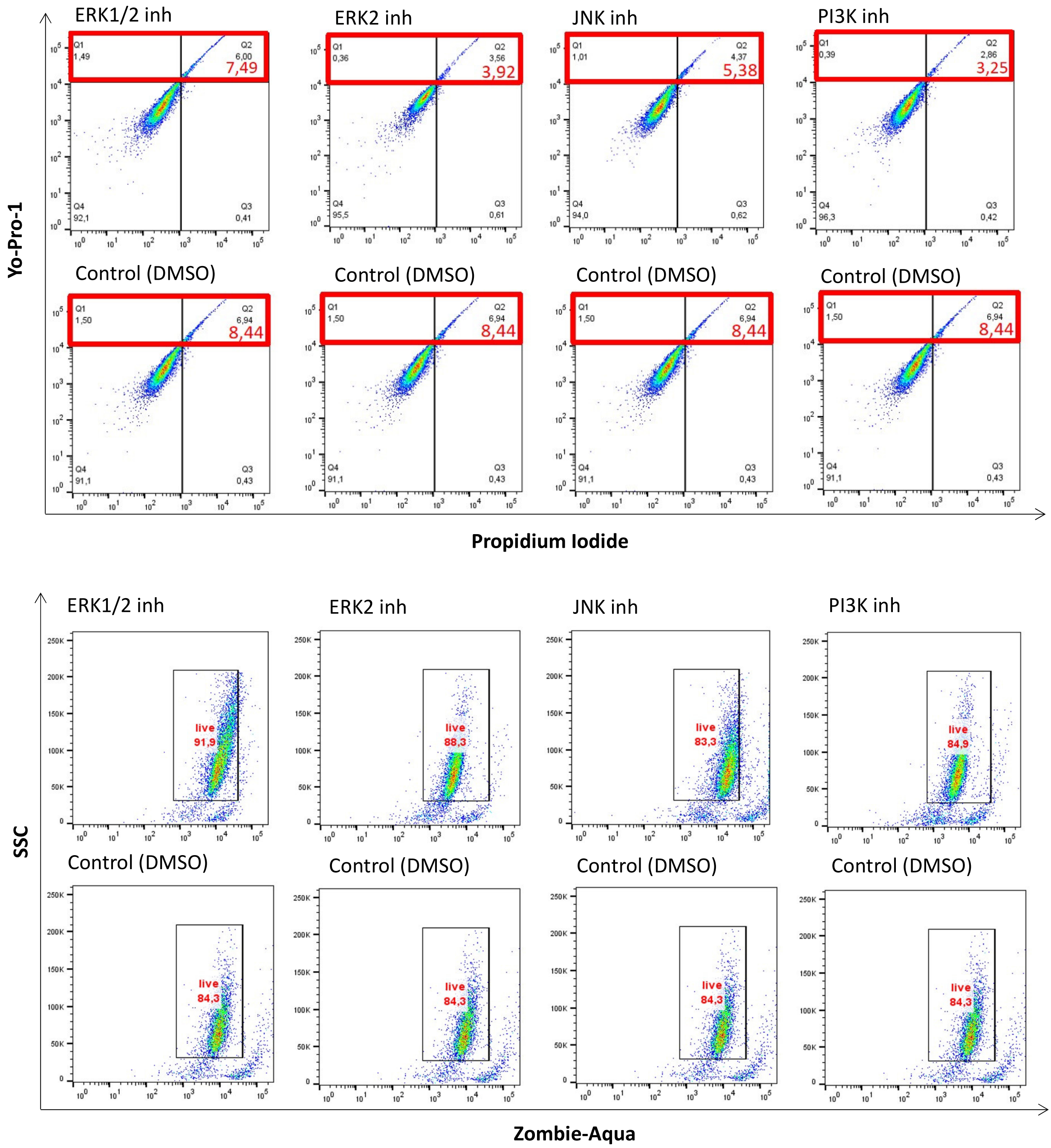

Supplementary Figure S6. The inhibitors employed in main Fig. 2 exert no cytotoxicity on cultured skin MC. Cultured skin-derived MCs were incubated with ERK1/2 inhibitor (inh), ERK2 inhibitor, JNK inhibitor, PI3K inhibitor, or with corresponding controls (solvent of each inhibitor) at the concentrations and preincubation times used for experiments and described in Methods. Each control is given below the respective inhibitor. YO-PRO-1/Propidium Iodide were stained to detect apoptosis, Zombie Aqua was used to quantify viability. The red frames indicate the total percentage of apoptotic cells given in as red numbers. Cells were measured by flow cytometry. 1 out of 2 experiments is shown. Note that viability is high and not compromised by any inhibitor.

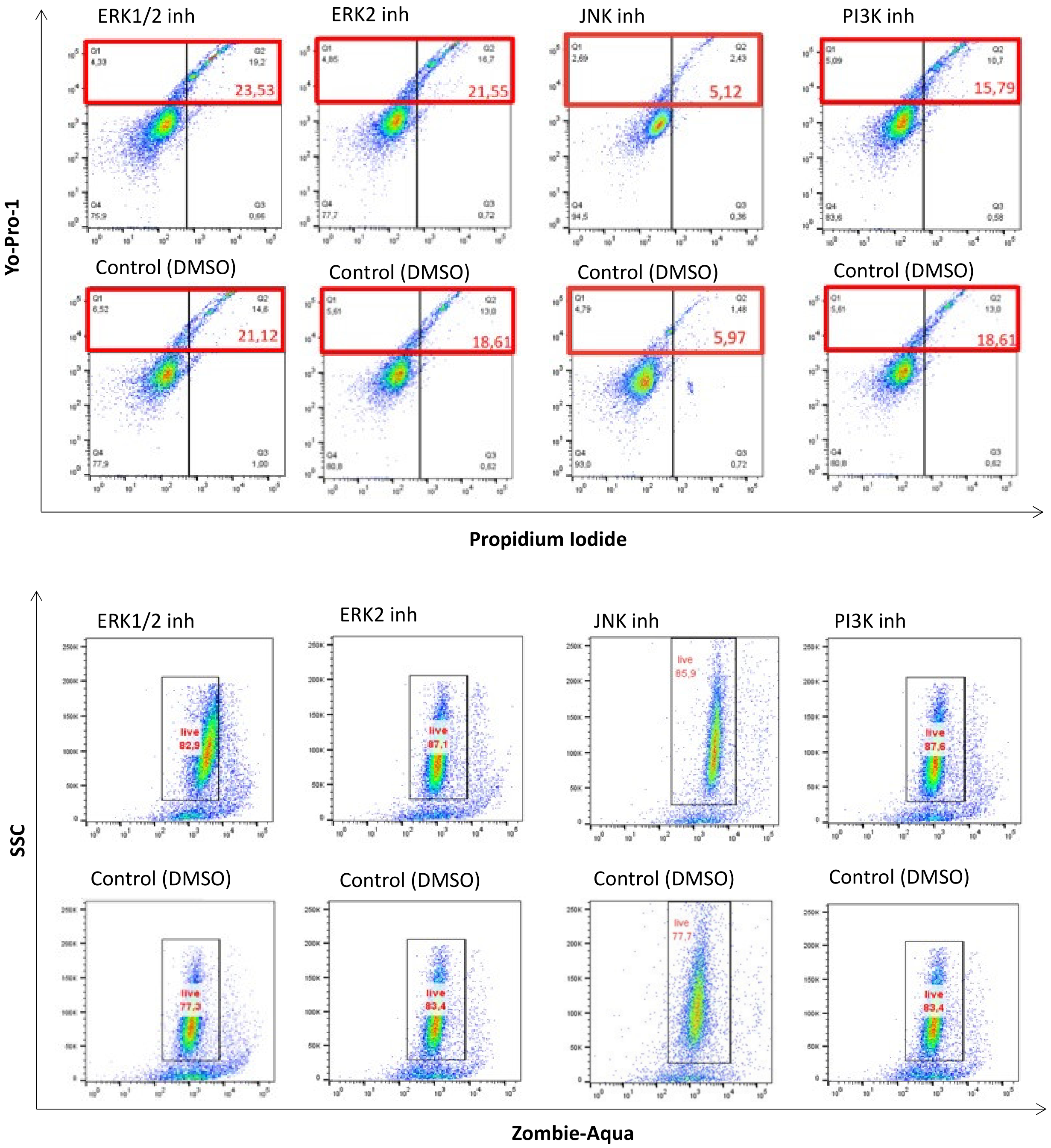

Supplementary Figure S7. The survival of *ex vivo* skin MCs is not perceptibly compromised by the inhibitors employed in main Fig. 2. *Ex vivo* skin MCs were incubated with ERK1/2 inhibitor (inh), ERK2 inhibitor, JNK inhibitor, PI3K inhibitor, or with corresponding controls (solvent of each inhibitor) at the concentrations and preincubation times used for experiments and described in Methods. Each control is given below the respective inhibitor. YO-PRO-1/Propidium Iodide were stained to detect apoptosis, Zombie Aqua was used to quantify viability. The red frames indicate the total percentage of apoptotic cells given in as red numbers. Cells were measured by flow cytometry. Results are pooled from 3 different experiments. Note that viability is high and not compromised by any inhibitor.

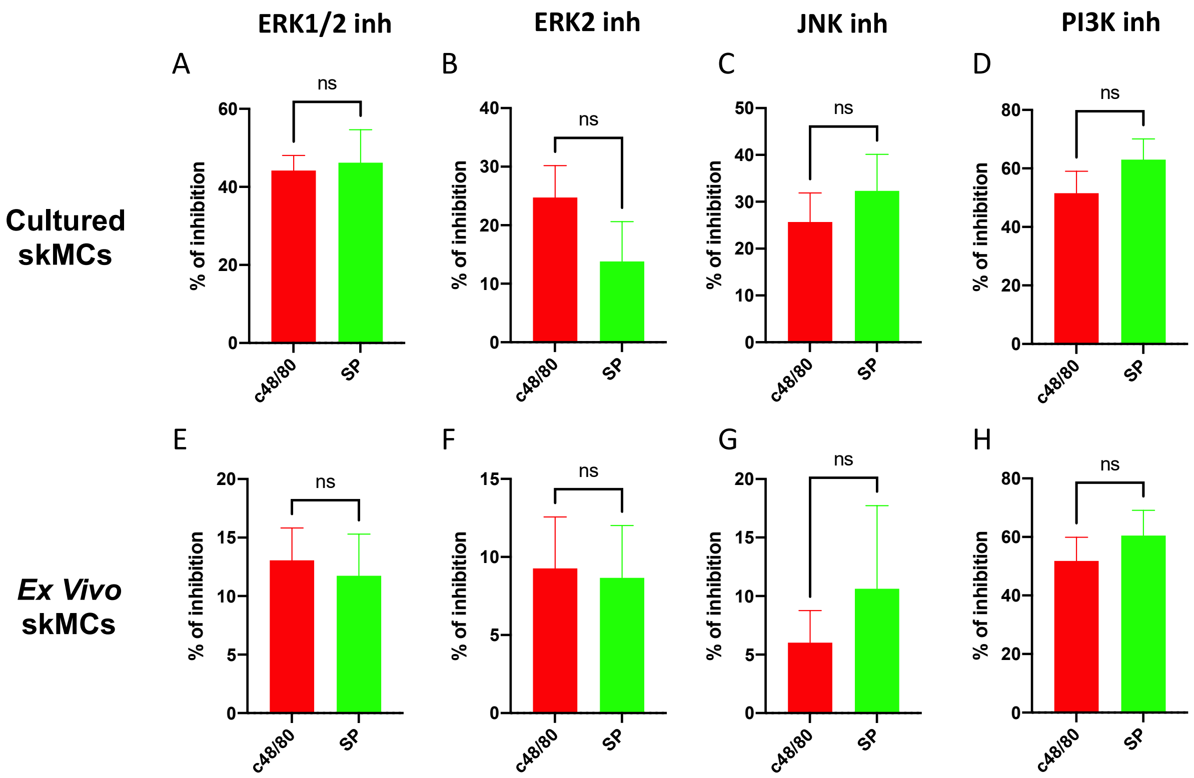

Supplementary Figure S8. Differences between c48/80 and SP from main Fig. 2. (A-D) Cultured or (E-H) *ex vivo* skin derived MCs were pre-treated with ERK1/2, ERK2, JNK or PI3K inhibitors (inh) and triggered by c48/80 or SP, β-hexosaminidase release was measured according to Fig 2. % inhibition (calculated as described in Methods) was compared between c48/80 and SP. The data are from 5-11 independent experiments, ns: not significant, skMCs: skin mast cells.

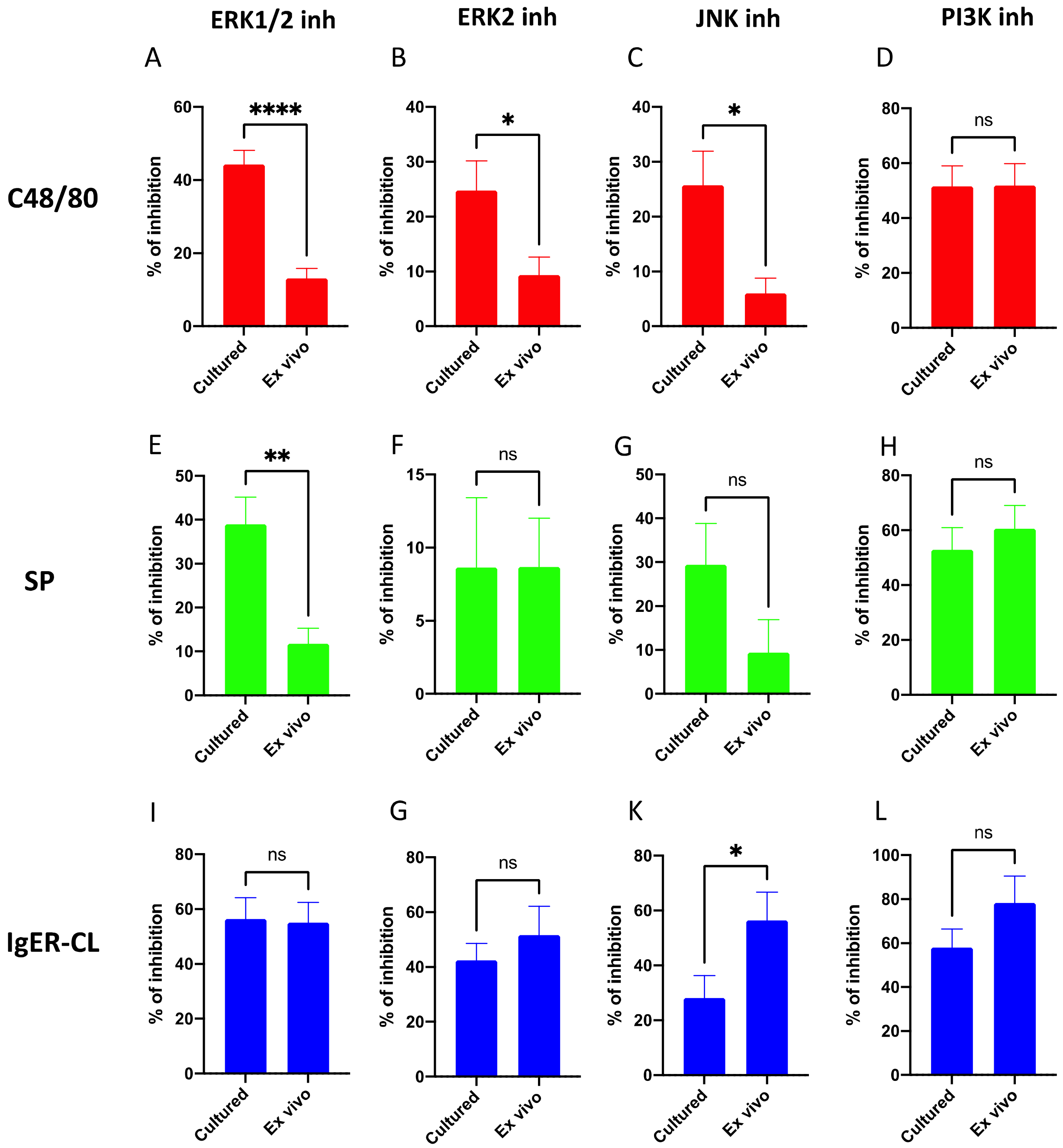

Supplementary Figure S9. Comparison between cultured and *ex vivo* skin MCs from main Fig. 2. Cultured and *ex vivo* skin MCs were pre-treated with ERK1/2, ERK2, JNK or PI3K inhibitors (inh) and stimulated by c48/80, SP or IgER-CL (cross-linking) according to Fig 2. β-hexosaminidase release was determined. % inhibition (calculated as described in Methods) for (A-D) c48/80, (E-H) SP or (I-L) IgER-CL was compared between the MC subsets. The data are from 5-11 independent experiments, ns: not significant, * $p < 0.05$; ** $p < 0.01$; **** $p < 0.0001$.

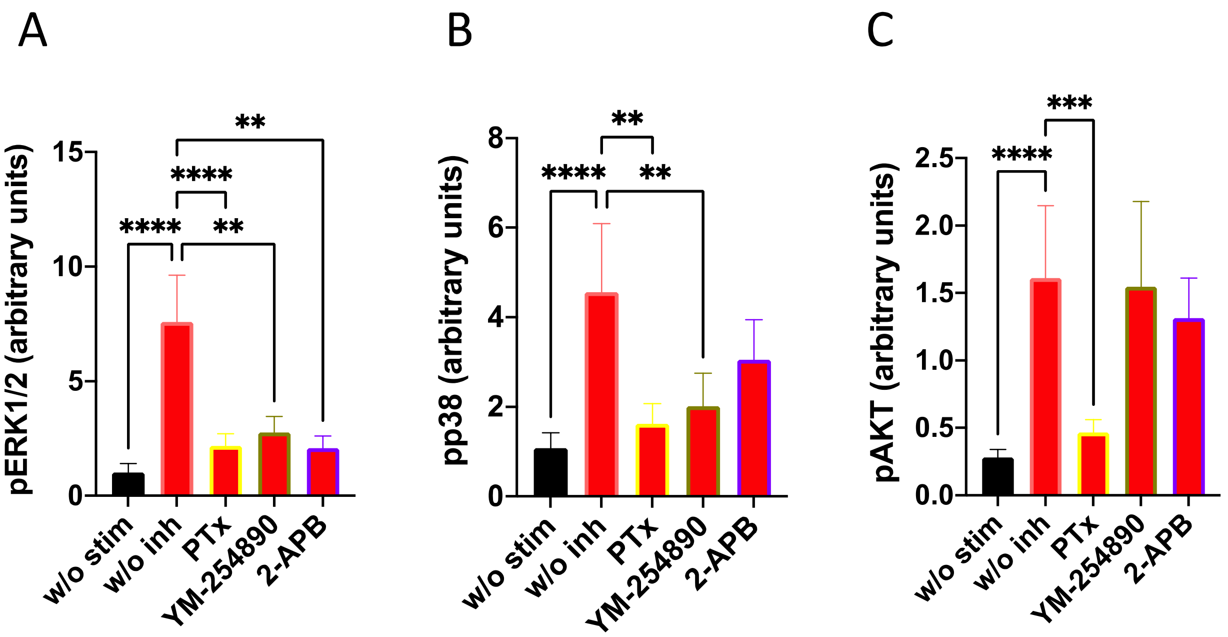

Supplementary Figure S10. c48/80 activated pERK depends on Gαi, Gαq, and Ca++, while pAKT exclusively requires the action of Gαi only - normalization against actinin. Cultured skin derived MCs were pretreated with PTX, YM-254890, 2-APB, or no inhibitor then stimulated with c48/80 for 1 min as in Main Fig. 3. Cells receiving no inhibitor or stimulus were the negative control (w/o stim). Vehicle controls showed no difference versus "w/o inh". Phosphorylation signals detected consecutively on the same membranes for (A) ERK1/2, (B) p38 and (C) AKT, quantified and normalized against actinin. Mean ± SEM of 16-18 independent experiments (individual cultures). ** $p < 0.01$, *** $p < 0.001$, **** $p < 0.0001$.

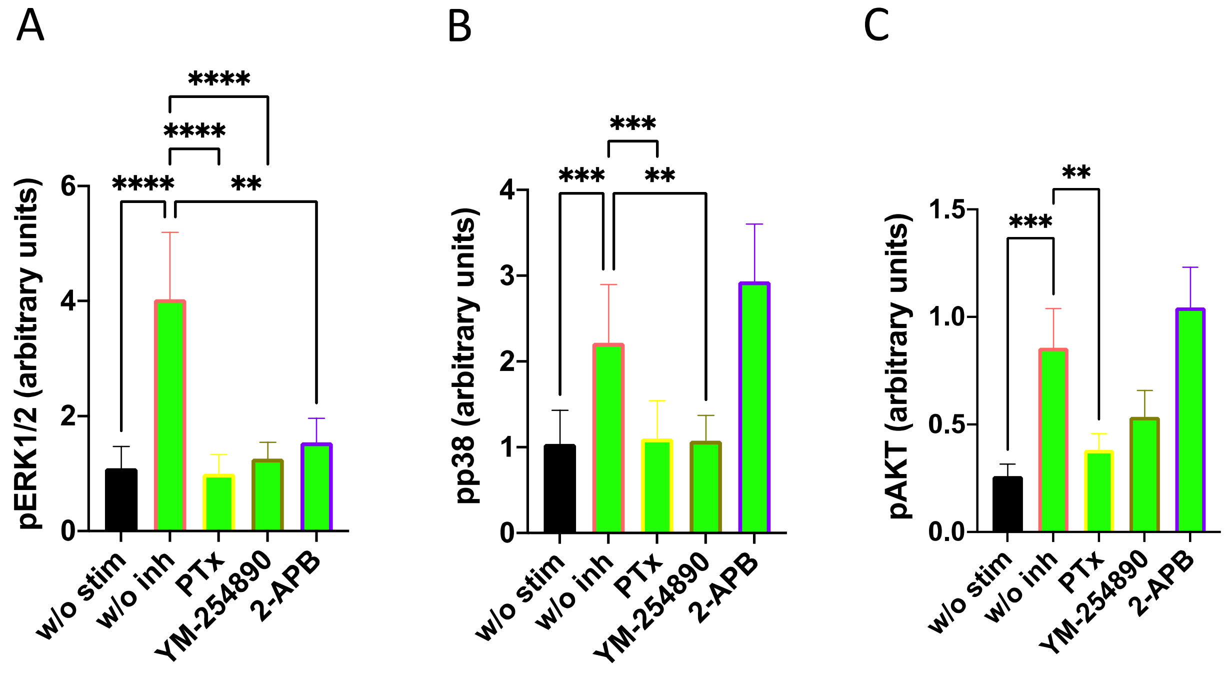

Supplementary Figure S11. Interconnections between early and later signals induced by SP are comparable to those elicited by c48/80 - normalization against actinin. MCs were pretreated with inhibitors then stimulated with SP as described in Main Fig. 4.

Signals of (A) pERK1/2, (B) pp38 and (C) pAKT were quantified and normalized with actinin. Vehicle controls showed no difference versus "w/o inh". The data are shown as Mean ± SEM of 15-20 independent experiments. ** $p < 0.01$, *** $p < 0.001$, **** $p < 0.0001$.

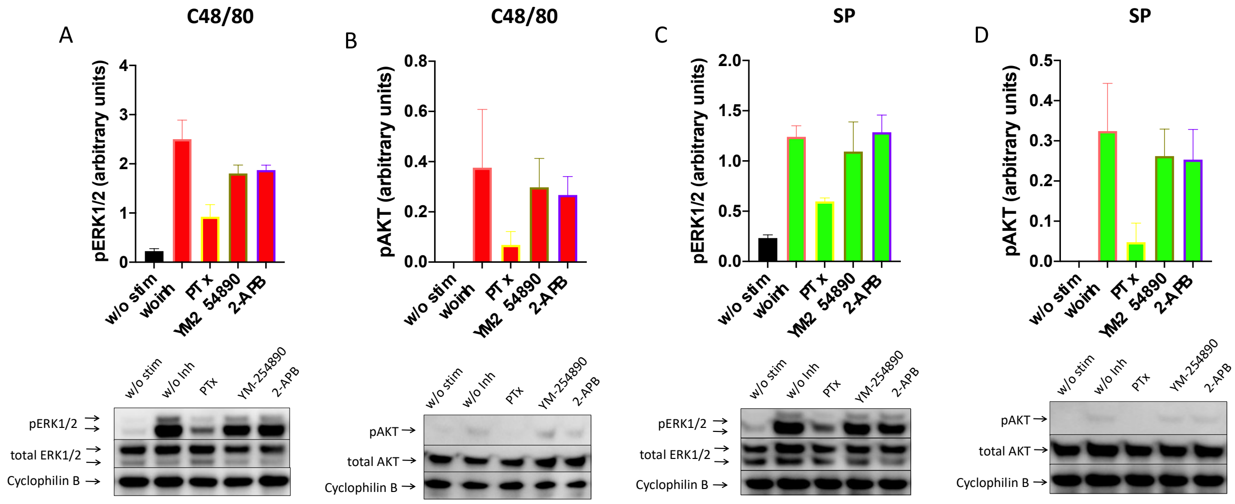

Supplementary Figure S12. Interconnections between early and later signals induced by c48/80 and SP in *ex vivo* MCs. *Ex vivo* MCs were pretreated with inhibitors then stimulated by c48/80 and SP as described in Main Figs. 3 and 4. Signals of A) and C) pERK1/2 and B) and D) pAKT were quantified and normalized to cyclophilin B as described in methods (upper panel). The data shown are Mean ± SEM of 3 independent experiments. Lower panel: Representative blots.

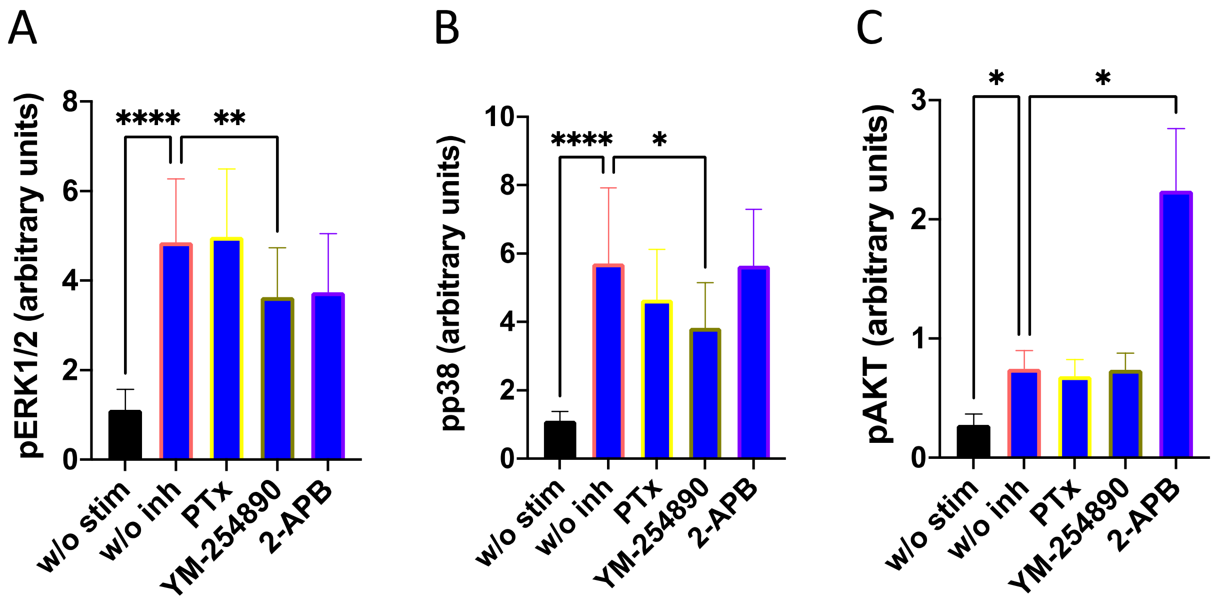

Supplementary Figure S13. Ca++ channel inhibition boosts AKT activation downstream of IgER-CL - normalization against actinin. MCs were pretreated with inhibitors then stimulated with AER-37 for FcεRI-aggregation (as described in main Fig. 5). Signals of (A) pERK1/2, (B) pp38 and (C) pAKT were quantified and normalized to actinin as described in methods. n = 11-12, Mean ± SEM. * $p < 0.05$, *** $p < 0.001$, **** $p < 0.0001$.

References

1. Murakami, T.; Suzuki, K.; Niyonsaba, F.; Tada, H.; Reich, J.; Tamura, H.; Nagaoka, I. MrgX2mediated internalization of LL37 and degranulation of human LAD2 mast cells. *Mol Med Rep* **2018**, *18*, 4951-4959, doi:10.3892/mmr.2018.9532.
2. Pundir, P.; Catalli, A.; Leggiadro, C.; Douglas, S.E.; Kulka, M. Pleurocidin, a novel antimicrobial peptide, induces human mast cell activation through the FPRL1 receptor. *Mucosal Immunol* **2014**, *7*, 177-187, doi:10.1038/mi.2013.37.
3. Aung, G.; Niyonsaba, F.; Ushio, H.; Kajiwara, N.; Saito, H.; Ikeda, S.; Ogawa, H.; Okumura, K. Catestatin, a neuroendocrine antimicrobial peptide, induces human mast cell migration, degranulation and production of cytokines and chemokines. *Immunology* **2011**, *132*, 527-539, doi:10.1111/j.1365-2567.2010.03395.x.
4. Naranjo, A.N.; Bandara, G.; Bai, Y.; Smelkinson, M.G.; Tobio, A.; Komarow, H.D.; Boyden, S.E.; Kastner, D.L.; Metcalfe, D.D.; Olivera, A. Critical Signaling Events in the Mechanoactivation of Human Mast Cells through p.C492Y-ADGRE2. *J Invest Dermatol* **2020**, *140*, 2210-2220 e2215, doi:10.1016/j.jid.2020.03.936.
5. Roy, S.; Ganguly, A.; Haque, M.; Ali, H. Angiogenic Host Defense Peptide AG-30/5C and Bradykinin B2 Receptor Antagonist Icatibant Are G Protein Biased Agonists for MRGPRX2 in Mast Cells. *J Immunol* **2019**, *202*, 1229-1238, doi:10.4049/jimmunol.1801227.
6. Manabe, Y.; Yoshimura, M.; Sakamaki, K.; Inoue, A.; Kakinoki, A.; Hokari, S.; Sakanaka, M.; Aoki, J.; Miyachi, H.; Furuta, K.; et al. 1-Fluoro-2,4-dinitrobenzene and its derivatives act as secretagogues on rodent mast cells. *Eur J Immunol* **2017**, *47*, 60-67, doi:10.1002/eji.201646536.
7. Morimoto, K.; Shirata, N.; Taketomi, Y.; Tsuchiya, S.; Segi-Nishida, E.; Inazumi, T.; Kabashima, K.; Tanaka, S.; Murakami, M.; Narumiya, S.; et al. Prostaglandin E2-EP3 signaling induces inflammatory swelling by mast cell activation. *J Immunol* **2014**, *192*, 1130-1137, doi:10.4049/jimmunol.1300290.
8. Subramanian, H.; Gupta, K.; Lee, D.; Bayir, A.K.; Ahn, H.; Ali, H. beta-Defensins activate human mast cells via Mas-related gene X2. *J Immunol* **2013**, *191*, 345-352, doi:10.4049/jimmunol.1300023.
9. Xiong, X.F.; Zhang, H.; Underwood, C.R.; Harpsoe, K.; Gardella, T.J.; Woldike, M.F.; Mannstadt, M.; Gloriam, D.E.; Brauner-Osborne, H.; Stromgaard, K. Total synthesis and structure-activity relationship studies of a series of selective G protein inhibitors. *Nat Chem* **2016**, *8*, 1035-1041, doi:10.1038/nchem.2577.
10. Arora, R.; Van Theemsche, K.M.; Van Remoortel, S.; Snyders, D.J.; Labro, A.J.; Timmermans, J.P. Constitutive, Basal, and beta-Alanine-Mediated Activation of the Human Mas-Related G Protein-Coupled Receptor D Induces Release of the Inflammatory Cytokine IL-6 and Is Dependent on NF-kappaB Signaling. *Int J Mol Sci* **2021**, *22*, doi:10.3390/ijms222413254.
11. Chompunud Na Ayudhya, C.; Roy, S.; Alkanfari, I.; Ganguly, A.; Ali, H. Identification of Gain and Loss of Function Missense Variants in MRGPRX2's Transmembrane and Intracellular Domains for Mast Cell Activation by Substance P. *Int J Mol Sci* **2019**, *20*, doi:10.3390/ijms20215247.
12. Ng, N.M.; Jiang, S.P.; Zhang, W. 2-Aminoethoxydiphenyl borate reduces degranulation and release of cytokines in a rat mast cell line. *Eur Rev Med Pharmacol Sci* **2012**, *16*, 1017-1021.
13. Lee, H.S.; Park, C.S.; Lee, Y.M.; Suk, H.Y.; Clemons, T.C.; Choi, O.H. Antigen-induced Ca2+ mobilization in RBL-2H3 cells: role of I(1,4,5)P3 and S1P and necessity of I(1,4,5)P3 production. *Cell Calcium* **2005**, *38*, 581-592, doi:10.1016/j.ceca.2005.08.002.
14. Chen, Y.C.; Chang, Y.C.; Chang, H.A.; Lin, Y.S.; Tsao, C.W.; Shen, M.R.; Chiu, W.T. Differential Ca(2+) mobilization and mast cell degranulation by FcepsilonRI- and GPCR-mediated signaling. *Cell Calcium* **2017**, *67*, 31-39, doi:10.1016/j.ceca.2017.08.002.
15. Schmid, E.; Gu, S.; Yang, W.; Munzer, P.; Schaller, M.; Lang, F.; Stournaras, C.; Shumilina, E. Serum- and glucocorticoid-inducible kinase SGK1 regulates reorganization of actin cytoskeleton in mast cells upon degranulation. *Am J Physiol Cell Physiol* **2013**, *304*, C49-55, doi:10.1152/ajpcell.00179.2012.
16. Schindl, R.; Kahr, H.; Graz, I.; Groschner, K.; Romanin, C. Store depletion-activated CaT1 currents in rat basophilic leukemia mast cells are inhibited by 2-aminoethoxydiphenyl borate. Evidence for a regulatory component that controls activation of both CaT1 and CRAC (Ca(2+) release-activated Ca(2+) channel) channels. *J Biol Chem* **2002**, *277*, 26950-26958, doi:10.1074/jbc.M203700200.

17. Ashmole, I.; Duffy, S.M.; Leyland, M.L.; Morrison, V.S.; Begg, M.; Bradding, P. CRACM/Orai ion channel expression and function in human lung mast cells. *J Allergy Clin Immunol* **2012**, *129*, 1628-1635 e1622, doi:10.1016/j.jaci.2012.01.070.
18. Chang, W.C.; Di Capite, J.; Singaravelu, K.; Nelson, C.; Halse, V.; Parekh, A.B. Local Ca2+ influx through Ca2+ release-activated Ca2+ (CRAC) channels stimulates production of an intracellular messenger and an intercellular pro-inflammatory signal. *J Biol Chem* **2008**, *283*, 4622-4631, doi:10.1074/jbc.M705002200.
19. Reinsprecht, M.; Rohn, M.H.; Spadinger, R.J.; Pecht, I.; Schindler, H.; Romanin, C. Blockade of capacitive Ca2+ influx by Cl- channel blockers inhibits secretion from rat mucosal-type mast cells. *Mol Pharmacol* **1995**, *47*, 1014-1020.
20. Hide, M.; Beaven, M.A. Calcium influx in a rat mast cell (RBL-2H3) line. Use of multivalent metal ions to define its characteristics and role in exocytosis. *J Biol Chem* **1991**, *266*, 15221-15229.
21. Wang, Z.; Franke, K.; Zuberbier, T.; Babina, M. Cytokine Stimulation by MRGPRX2 Occurs with Lower Potency than by FcepsilonRI Aggregation but with Similar Dependence on the Extracellular Signal-Regulated Kinase 1/2 Module in Human Skin Mast Cells. *J Invest Dermatol* **2021**, doi:10.1016/j.jid.2021.07.153.
22. Babina, M.; Wang, Z.; Franke, K.; Zuberbier, T. Thymic Stromal Lymphopoietin Promotes MRGPRX2-Triggered Degranulation of Skin Mast Cells in a STAT5-Dependent Manner with Further Support from JNK. *Cells* **2021**, *10*, doi:10.3390/cells10010102.
23. Franke, K.; Wang, Z.; Zuberbier, T.; Babina, M. Cytokines Stimulated by IL-33 in Human Skin Mast Cells: Involvement of NF-kappaB and p38 at Distinct Levels and Potent Co-Operation with FcepsilonRI and MRGPRX2. *Int J Mol Sci* **2021**, *22*, doi:10.3390/ijms22073580.
24. Wang, Z.; Guhl, S.; Franke, K.; Artuc, M.; Zuberbier, T.; Babina, M. IL-33 and MRGPRX2-Triggered Activation of Human Skin Mast Cells-Elimination of Receptor Expression on Chronic Exposure, but Reinforced Degranulation on Acute Priming. *Cells* **2019**, *8*, doi:10.3390/cells8040341.
25. Babina, M.; Wang, Z.; Franke, K.; Guhl, S.; Artuc, M.; Zuberbier, T. Yin-Yang of IL-33 in Human Skin Mast Cells: Reduced Degranulation, but Augmented Histamine Synthesis through p38 Activation. *J Invest Dermatol* **2019**, *139*, 1516-1525 e1513, doi:10.1016/j.jid.2019.01.013.
